# A translational view of airway epithelial dysfunction in COPD

**DOI:** 10.1183/16000617.0110-2025

**Published:** 2025-12-17

**Authors:** Christopher E. Brightling, Mona Bafadhel, MeiLan K. Han, Jean-Francois Papon, Klaus F. Rabe, Paola Rogliani, Dave Singh

**Affiliations:** 1 Institute for Lung Health, National Institute for Health and Care Research, Leicester Biomedical Research Centre, University of Leicester, Leicester, UK; 2 King's Centre for Lung Health, School of Immunology and Microbial Sciences, Faculty of Life Sciences and Medicine, King's College London, London, UK; 3 Division of Pulmonary and Critical Care, University of Michigan, Ann Arbor, MI, USA; 4 Otolaryngology Department, Cochlear Implant Center, Bicêtre Hospital, Greater Paris University Hospitals, Paris-Saclay University, Kremlin-Bicêtre, France; 5 LungenClinic Grosshansdorf, Grosshansdorf, Germany; 6 Airway Research Center North, German Center for Lung Research, Grosshansdorf, Germany; 7 Christian-Albrechts University of Kiel, Kiel, Germany; 8 Unit of Respiratory Medicine, Department of Experimental Medicine, University of Rome Tor Vergata, Rome, Italy; 9 Manchester University NHS Foundation Trust, University of Manchester, Manchester, UK; 10 Medicines Evaluation Unit, Manchester, UK

## Abstract

COPD is a heterogeneous, progressive inflammatory airway disease, with patients presenting with a wide variety of symptoms, comorbid conditions and underlying pathophysiology. Smoking is a major contributing factor to disease burden, alongside other environmental and patient-related risk factors. Airway inflammation is consistently present in COPD and is implicated in disease pathogenesis and progression. The airway epithelium functions as an active physiochemical barrier, protecting the lungs from pathogens and airborne environmental triggers, and as an immune organ that coordinates immunological activity in response to pollutant, bacterial, viral or allergen exposure. Inhalation of cigarette smoke and other airborne triggers can damage bronchial epithelial cells, leading to exaggerated inflammatory responses and airway remodelling. Airway inflammation in COPD, including neutrophilic and eosinophilic phenotypes, is mediated by the epithelium and epithelial cell-derived cytokines. Improving our understanding of epithelial-related inflammation in COPD is essential for the identification of novel biomarkers, stratification of patients, development of targeted therapeutics and creation of personalised treatment strategies. Here, we review the current understanding of the role of the airway epithelium in COPD pathogenesis, providing an overview of the pathological changes to the epithelium and the role of the epithelial-derived cytokines in driving different inflammatory phenotypes. We then consider biomarkers related to epithelial function in COPD and discuss how the epithelium might be targeted by novel COPD therapies.

## Shareable abstract

**An enhanced understanding of epithelial-related inflammation in COPD is critical for discovering novel biomarkers, stratifying patients, creating targeted therapies and developing personalised treatments** https://bit.ly/3J2k1FI

## Introduction

COPD is a growing global health crisis and is one of the top three leading causes of death worldwide, accounting for ~3 million deaths annually [1]. COPD is a heterogeneous inflammatory airway disease, with patients presenting with a wide variety of symptoms, comorbid conditions and underlying pathophysiology [2, 3].

Although smoking is a major contributor to the development of COPD and to the disease burden, COPD risk factors are multifactorial [4]. Environmental risk factors include smoking, occupational exposure and pollution (*e.g.* biomass exposure and pathogens) [5, 6]. Patient-related risk factors such as lung development and function, comorbidities, genetics and age also have a key role [2].

Common COPD symptoms include dyspnoea, chronic cough, exercise intolerance and exacerbations [2]. These symptoms are coupled with changes in airway pathophysiology, characterised by inflammation, airway remodelling and tissue destruction [2]. The heterogeneity of COPD is driven by different molecular mechanisms, or endotypes, and is influenced by complex host–environment interactions at the protein-to-cell and tissue-to-organ levels. Together, these interactions determine the clinical phenotypes of COPD [2, 5].

Airway inflammation is consistently present in COPD and is implicated in the pathogenesis and progression of the disease. Inhalation of cigarette smoke and other airborne environmental triggers can damage bronchial epithelial cells and lead to chronic pro-inflammatory responses and airway remodelling [5]. Airway inflammation is mediated by the epithelium and epithelial-derived cytokines [7, 8]. The inflammatory response in COPD involves both innate and adaptive immunity; the most common inflammatory phenotype is neutrophilic inflammation, which is driven by type 1 and type 3 immune responses [5, 8]. However, high eosinophilic inflammation is also observed alongside this abnormal innate immunity in a subgroup of patients with COPD, reflecting the complexity of the disease [5].

Increasing our understanding of epithelial-related inflammation in COPD is essential for the identification of novel biomarkers, stratification of patients, development of targeted therapeutics and creation of personalised treatment strategies [7, 8]. This review provides an overview of the role of the airway epithelium in COPD pathogenesis, detailing the pathological changes to the epithelium and the role of the epithelial-derived cytokines in driving different inflammatory phenotypes. We consider biomarkers related to epithelial function in COPD and discuss how the epithelium might be targeted by novel COPD therapies.

## Structural changes to the epithelium in COPD

### Airway epithelial structure and function

**FIGURE 1:**
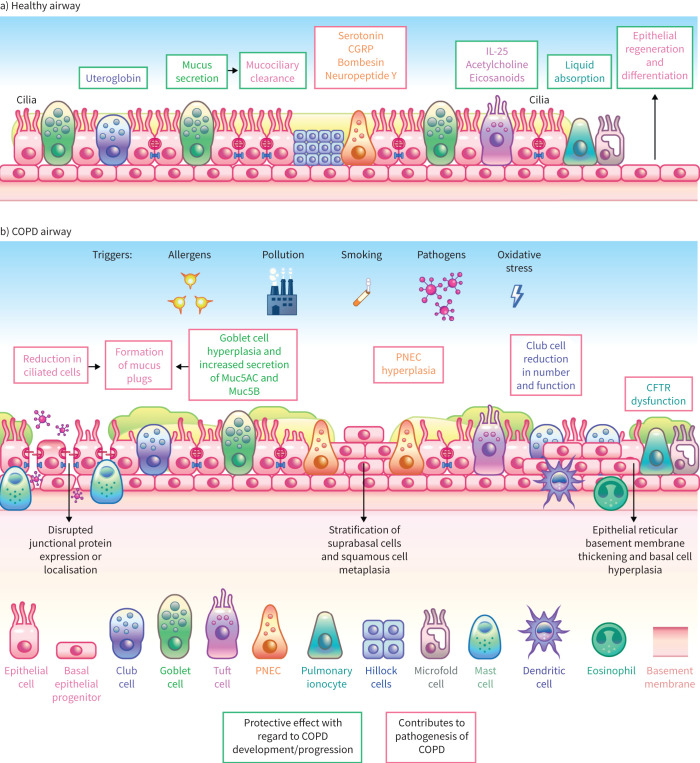
The airway epithelium in a) healthy individuals and b) patients with COPD. The different cells that comprise the airway epithelium play functionally distinct roles in a) health and b) disease. In the healthy airway, basal cells are the principal stem cells of the airway, facilitating epithelial regeneration. In disease, respiratory epithelial cells can contribute to different illnesses, such as COPD. CGRP: calcitonin gene-related peptide; IL: interleukin; PNEC: pulmonary neuroendocrine cell; CFTR: cystic fibrosis transmembrane conductance regulator. Information from [10, 11].

The airway epithelium acts as an active physiochemical barrier, protecting the lungs from pathogens, pollutants and other airborne environmental triggers, which can induce inflammatory responses and airway remodelling [9]. Alongside its function as a physical barrier, the epithelium acts as an immune organ in response to bacterial, viral or environmental exposure and communicates with the immune system to maintain respiratory health (figure 1a and table 1) [10, 36].

**TABLE 1:** Roles of the airway epithelial cells

	Upper airway	Lower airway	Role in disease
**Basal cells**	Include both quiescent stem cells and dividing progenitor cells, and are firmly attached to neighbouring epithelial cells, anchoring the epithelium to the basal membrane Upon injury, basal cells are activated and detach from the basal membrane, before migrating towards the epithelial culprit and forming a provisional barrier [12] Depending on Notch signalling regulation and following epithelial damage, basal cells can function as precursors for ciliated cells (upon suppression of Notch signalling), secretory cells (promoted by high levels of Notch signalling) [13, 14] or other specialised epithelial cells (such as ionocytes and tuft/brush cells) [15, 16]	The proportion of basal cells in the airway epithelium is highest in the large airways and progressively decreases going down the tracheobronchial tree, representing an average of 34% in the trachea, 27% in the large airways and 10% in the small airways [17]	Hyperplasia of basal cells is regarded as one of the earliest structural abnormalities associated with COPD [17] In COPD airways, increased numbers of basal cells show deregulated differentiation, which contribute to ciliary dysfunction and mucus hyperproduction [18]
**Ciliated cells**	Exhibit large numbers of cilia on the apical side, which are required for the mucociliary clearance of the airway cell surface [12]	Are present in lower airways, but at a lower frequency than in the upper airway [19]	Reduced ciliary beat frequency, shortened cilia or ciliary depletion are features of the airway epithelium of patients with COPD [18, 20], asthma and allergic rhinitis [12, 21]
**Secretory cells**			
Goblet cells	Contain vesicles with tightly packed mucin granules and surfactant proteins Their primary role is to secrete mucins onto the internal surface of the airway so that environmental molecules can be trapped [11]	Are present in lower airways, but at a lower frequency than in the upper airway [19]	In health, there is a fine equilibrium between the production and clearance of mucins Excessive goblet cell differentiation disturbs the balance of Muc5AC and Muc5B (the major mucin proteins in the airways), which is increased in patients with COPD [18, 20, 22]
Club cells	Are not present in the upper airway [23]	Secretory nonciliated cells of the small airways that release substances required for airway protection and repair [11], including protease inhibitors, surfactant-stabilising proteins, antimicrobial peptides and mediators of the innate immune response [11, 23]	Club cell numbers are decreased in patients with COPD [20]
**Suprabasal cells**	Are located between the basal and luminal cells of the airway epithelium [24] Potentially act as precursors to secretory cells, but possess a limited ability to proliferate [12, 25, 26] Present in region-specific subclusters between the upper and lower airways [27]	Are present in lower airways [27]	
**Ionocytes**	Differentiate from basal cells, with Notch signalling and *FOXI1* expression driving the production of ionocytes [16] Are a major source of CFTR, suggesting a potential crucial role in regulating epithelial barrier function [16]	The distribution of ionocytes, and consequently the expression of CFTR may vary along the proximodistal axis of the airway tree, being less abundant in lower airways [28]	Ionocytes show co-enrichment of the proton-secreting V-ATPase, suggesting a role in regulating luminal pH and mucus viscosity that could be relevant for the pathogenesis of cystic fibrosis [16, 24] Evidence of CFTR dysfunction has been observed in patients with COPD [29] *In vitro* data suggest that opposing effects on CFTR in secretory cells and ionocytes regulate chloride secretion and absorption and, consequently, liquid secretion [30]
**Neuroendocrine cells**	Are strategically positioned at airway branching points where allergens and other harmful substances accumulate These cells serve as airway chemoreceptors that monitor airway status and signal this status to other lung cells, or to the brain through synapses with the nervous system [31] In mouse models, tracheal and laryngeal neuroendocrine cells protect the airways by releasing ATP to activate purinoreceptive sensory neurons that initiate swallowing and expiratory reflexes [32]	Are present in lower airways, but are more abundant in the larynx, where they collaborate with laryngeal taste cells to provide layers of protection with expiratory and coughing-like reflexes, preventing noxious stimuli from reaching the lungs [32]	Increased numbers of neuroendocrine cells have been reported in patients with COPD and other lung diseases [33]
**Tuft/brush cells**	Also known as solitary chemosensory cells, tuft/brush cells potentially play a role in regulating the innate immune response in the airways [34]	A higher proportion of chemosensory cells are found in the distal bronchi than in the upper airway [34]	Tuft/brush cells regulate type 2 immunity and serve as a reservoir of epithelial IL-25, which is often seen in patients with allergic asthma and CRSwNP [12] These cells have been reported to sense virulence-associated formyl peptides and to release acetylcholine to activate tracheal mucociliary clearance; formylated bacterial peptides have been detected in sputum from patients with COPD [35]

CFTR: cystic fibrosis transmembrane conductance regulator; V-ATPase: vacuolar-type ATPase; IL: interleukin; CRSwNP: chronic rhinosinusitis with nasal polyps.

In the large airways, the airway is pseudostratified and primarily composed of five functionally and structurally distinct cell types (basal cells, ciliated cells, secretory (goblet and club) cells and suprabasal cells), alongside rarer cell types such as ionocytes, neuroendocrine cells and tuft/brush cells (table 1) [11, 12, 37]; in the small airways (<2 mm in diameter), the cell types become more limited to columnar and/or cuboidal [37]. In a healthy adult, the composition of epithelial cell populations changes by airway level. As the airways branch from large to small airways, there is a decrease in the proportion of cartilage cells and submucosal glands, and the emergence of secretory cells (table 1) [37, 38].

Basal cells, which include both quiescent stem cells and dividing progenitor cells, function as precursors for ciliated and secretory cells following damage [12]. Using numerous motile cilia, ciliated cells clear material trapped in the mucus from the airway cell surface [12]. Goblet cells are simple columnar epithelial cells that are responsible for the secretion of mucin and other surfactant proteins onto internal airway surfaces, thereby trapping environmental molecules [12]. Club cells are cuboidal in structure and release substances required for airway protection and repair, including protease inhibitors, surfactant-stabilising proteins, antimicrobial peptides and mediators of the innate immune response [11]. Suprabasal cells are located between the basal and luminal cells of the airway epithelium. They exhibit ultrastructural features similar to basal cells, but possess a limited ability to proliferate [24]. The permeability of the airway epithelium is regulated by junctional complexes, including adherens junctions, tight junctions, desmosomes and gap junctions [18]. The integrity of these constituent cells and their adhesions are vital to the physical barrier function of the epithelium [18].

### Epithelial remodelling in COPD

Exposure to cigarette smoke, oxidative stress and infection can trigger cycles of persistent, repeated injury, impaired epithelial repair and premature epithelial senescence, which cause chronic airway inflammation and remodelling. This leads to histological, cellular and molecular changes in the airways of patients with COPD that can ultimately drive fibrosis and alveolar destruction (emphysema) (figure 1b and table 1) [6, 18].

Airway remodelling in COPD includes ciliated cell dedifferentiation, goblet cell hyperplasia, basal cell hyperplasia and metaplasia, thickening and fibrosis of the subepithelial matrix and increased airway smooth muscle mass below the epithelium [6, 18]. Transmission electron microscopy of bronchial epithelial brushings showed a loss of cilia in patients with COPD *versus* healthy individuals [39]. Other ultrastructural changes identified included cell extrusion and cytoplasmic blebs [39].

Hyperplasia of airway epithelial basal cells is regarded as one of the earliest structural abnormalities associated with COPD [17]. Alongside this increase in basal cell number, airway epithelial basal cells appear to be functionally impaired and club cell numbers are decreased in COPD [20]. An inability to form a fully differentiated, structurally stable airway epithelium may underlie the decreased host defence function and barrier integrity of the airway epithelium observed in COPD [17]. In *ex vivo* cultures of small airway epithelial basal cells from patients with COPD who smoked, basal cells had a limited ability to regenerate a fully differentiated epithelium [40].

Chronic exposure to cigarette smoke is a key risk factor for COPD and has been shown to cause several changes in the airway epithelium, including a reduction in epithelial cell number, an increase in goblet cell number and a shortening of cilia [18]. Alongside a decrease in cilia number, the function of cilia may also be impaired in COPD. Nasal brushings of ciliated cells from patients with COPD showed a significant decrease in ciliary beat frequency *versus* those from healthy nonsmokers [41].

Goblet cell hyperplasia is also observed in patients with COPD and may lead to mucus hypersecretion [18, 42]. Expression of the primary mucins MUC5AC and MUC5B is increased in patients with COPD [43]. Together, ciliary dysfunction and mucus hypersecretion lead to mucus plugging and airway obstruction, which are associated with clinical symptoms of the disease, including a decrease in forced expiratory volume in 1 s (FEV_1_) and an increased risk of exacerbations [44].

Small airway disease is a recognised feature of COPD, with patients demonstrating marked epithelial remodelling, increased thickness of the airway wall and reduced flow velocity [38, 45, 46], which can lead to increased exposure to airborne particles [38]. Notably, the increased peripheral airway resistance and obstruction reported in patients with COPD can be explained by the narrowing and obliteration of small conducting airways, independently increasing the severity of disease before the onset of emphysematous destruction [45, 46].

Regulation of airway epithelial barrier permeability is impaired in COPD in response to stressors, including cigarette smoke, oxidative stress, viral infection and acute inflammation [18]. Reduced expression and mislocalisation of tight junction proteins, including occludin and zonula occludens-1, is observed in the epithelial cells of patients with COPD [18, 47]. Smoking also upregulates the expression of the epidermal growth factor (EGF) family members EGF and amphiregulin [17]. These proteins function through the EGF receptor (EGFR), which is highly expressed on human airway basal cells [48]. Chronic EGF stimulation *in vitro* induces squamous cell metaplasia, basal cell hyperplasia, goblet cell hyperplasia and cilia shortening [17].

Furthermore, cigarette smoke, inflammation, reactive oxygen species and bacterial byproducts can cause dysfunction of the cystic fibrosis transmembrane conductance regulator (CFTR) [29]. CFTR is a member of the ATP-binding cassette transporter superfamily and functions as a chloride and bicarbonate anion channel, facilitating the active transport of salt and water across epithelial surfaces [29]. The role of CFTR in cystic fibrosis pathogenesis is well established, and evidence of CFTR dysfunction has also been observed in COPD [29]. CFTR is localised to the apical membrane of epithelial cells, with the highest expression detected in the secretory and basal cells of the epithelium [49]. CFTR dysfunction has been observed in the upper and lower airways of patients with COPD and may contribute to disturbed epithelial surface liquid homeostasis, mucus plugging, pathogen colonisation and inflammation [29].

### Comparison of the structural changes to the airway epithelium between COPD and asthma

Similar to COPD, dysregulation of the airway epithelial barrier function is central to the progression of asthma [20]. Both diseases share several features, including altered apicobasal polarisation, goblet cell hyperplasia, disrupted junctional protein expression or localisation, as well as reduced basal-to-apical transcytosis of immunoglobulins [20]. Epithelial reticular basement membrane thickening is observed in both diseases, although this occurs to a greater extent in asthma than in COPD [18, 20].

Features typically associated with COPD but not with asthma include altered basal cell differentiation, a reduction in ciliated cells and squamous cell metaplasia, which are factors associated with altered mucociliary clearance of pathogens [20]. Emphysema, characterised by alveolar destruction and small airway obstruction, is another clinical manifestation of COPD that is not observed in asthma [18]. At the cellular level, aberrant inflammatory and oxidative stress responses drive alveolar epithelial cell death and impaired re-epithelialisation, ultimately leading to alveolar destruction and development of emphysema in COPD [50, 51].

## Role of epithelial-derived cytokines in COPD pathogenesis

**FIGURE 2:**
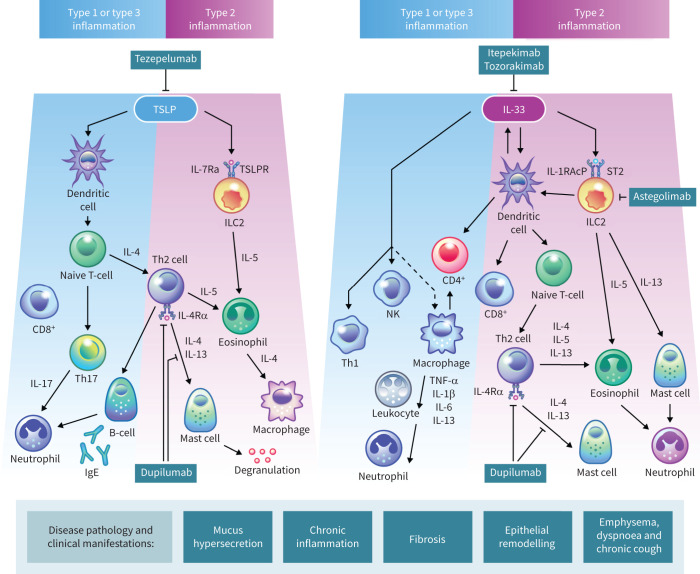
Role of epithelial-derived cytokines in the inflammatory cascade in COPD, including an overview of cytokine-targeting treatments that are in development for COPD. Upon epithelial damage in COPD, alarmins interleukin (IL)-33, IL-25 (not depicted here) and thymic stromal lymphopoietin (TSLP) are released from epithelial cells. TSLP promotes airway inflammation through binding to its receptor (TSLPR), which is present on several immune cells including type 2 innate lymphoid cells (ILC2s), dendritic cells, type 2 (T2) and regulatory T (Treg) cells, eosinophils and macrophages. TSLP has a broad effect, resulting in the production of cytokines typically associated with a T2 response, while also affecting mast cells, type 1 T-helper (Th1) cells, and other cells that result in the production of both T2 and non-T2 cytokines. IL-33 promotes inflammation through binding to its receptor serum-stimulated (ST)2 (IL-1 receptor-like 1 (IL-1R1)), which is present on various cells including neutrophils, eosinophils, macrophages, basophils and mast cells, leading to the production of both T2 and non-T2 cytokines. The released cytokines lead to the activation of various immune cells, contributing to the pathobiology of COPD. The monoclonal antibody dupilumab (anti-IL-4 receptor-α (IL-4Rα)) inhibits T2 inflammatory cytokines, whereas tezepelumab (anti-TSLP), astegolimab (anti-ST2), itepekimab (anti-IL-33) and tozorakimab (anti-IL-33) block epithelium-derived alarmins or their receptors on target immune cells. CD: cluster of differentiation; Ig: immunoglobulin; IL-1RAcP: IL-1 receptor accessory protein; NK: natural killer; TNF: tumour necrosis factor. Reproduced and modified from [11, 52, 53], with permission.

Alongside structural changes to the epithelium, damage to bronchial epithelial cells triggers the release of epithelial alarmin cytokines: interleukin (IL)-33, thymic stromal lymphopoietin (TSLP) and IL-25. These epithelial-derived cytokines play a key role in the initiation of a cascade of immune responses driving inflammation and structural changes that contribute to the clinical features of COPD (figure 2) [8, 52, 54].

### IL-33

IL-33 is constitutively expressed in the nuclei of epithelial and endothelial cells [7] and is released following viral infection, oxidative stress, exposure to allergen proteases or fungal allergens, or pyroptosis [55–60]. Upon release, IL-33 targets many different cell types, including those involved in type 1 immunity, such as type 1 T-helper (Th)1 cells, natural killer (NK) and NK T-cells, cluster of differentiation (CD)8^+^ T-cells, macrophages and neutrophils. IL-33 also targets cells involved in type 2 (T2) immune responses, including dendritic cells, T2 innate lymphoid cells (ILC2s), CD4^+^ Th2 cells, eosinophils, mast cells and basophils [7, 61, 62].

IL-33 is released in a reduced form that signals *via* serum stimulated (ST)2 [63]. ST2 pathway activation results in inflammatory cell recruitment and the release of a broad range of cytokines, including the T2 cytokines IL-4, IL-5 and IL-13 [7, 52]. The downstream inflammatory effects of these cytokines include the induction of B-cell immunoglobulin (Ig)E production and the activation of eosinophils and mast cells [5]. IL-33 can also signal through a pathway that is distinct from ST2-mediated inflammation. IL-33 becomes rapidly oxidised in the extracellular environment and signals *via* a complex consisting of the receptor for advanced glycation end products (RAGE) and EGFR [64]. IL-33 signalling through RAGE-EGFR drives airway epithelial remodelling, decreasing epithelial barrier function and increasing mucin production [64].

In the context of COPD, IL-33 might play a role in pathogen-induced exacerbations of the disease [65]. In a mouse model, smoke exposure reduced ST2 expression on ILC2s while increasing ST2 expression on macrophages and NK cells, thereby modifying IL-33 responsiveness within the lung. Upon viral infection, elevated local IL-33 levels significantly enhanced type 1 (T1) pro-inflammatory responses by synergistically modulating macrophage and NK cell function. Furthermore, mice lacking IL-33 were protected from the smoke-induced heightened inflammatory responses to viral infection, suggesting a potential IL-33 mediator role in acute COPD exacerbations [65].

Additionally, high IL-33 expression has been observed in the airways of patients with very severe COPD [65–68]. Furthermore, expression of IL-33 was significantly correlated with smoking pack-years in a cohort of healthy individuals and patients with COPD [67]. However, among patients with COPD, current smokers had reduced overall IL-33 levels *versus* former smokers, potentially mediated by a decrease in basal epithelial cell numbers caused by cigarette smoke exposure [69]. The number of exacerbations was significantly higher in patients with high plasma IL-33 levels than in those with low levels [67]. There is also evidence that IL-33 expression is related to eosinophilic inflammation in COPD, with increased serum and sputum IL-33 levels observed in patients with COPD with sputum eosinophilia (sputum eosinophil counts of >3%) *versus* those without sputum eosinophilia [70].

### TSLP

TSLP has also been linked to COPD pathogenesis [71]. Despite sharing some signalling pathways that promote T2 inflammation, TSLP and IL-33 have distinct receptor interactions and downstream effects, which reflects the complexity of immune regulation in the airway epithelium during COPD pathogenesis [7, 52, 72]. Epithelial cells are the primary source of TSLP in homeostatic and inflammatory conditions. However, immune cells also express TSLP, including dendritic cells, basophils and mast cells [7, 52]. Epithelial cell expression and release of TSLP increases in response to allergen proteases, inflammatory cytokines, mechanical injury and infections [7, 52].

TSLP mediates both T2 and non-T2 inflammation, acting on a broad range of cells, including eosinophils, basophils, mast cells, airway smooth muscle cells, ILC2s, dendritic cells, platelets, sensory neurons and macrophages [52]. For example, TSLP-activated dendritic cells induce naive CD4^+^ T-cell proliferation and Th2-cell differentiation, stimulating the production of T2 cytokines (IL-4, IL-5 and IL-13) to drive an inflammatory response [72]. Although TSLP is primarily regarded as a promoter of T2 inflammation, there is also evidence that it has a role in regulating type 1 chemokines, including C-X-C motif chemokine ligand (CXCL)10, in patients with COPD [73].

TSLP expression appears to be upregulated in patients with COPD *versus* healthy individuals [71, 73–75]. In addition, epithelial TSLP expression was shown to be increased in current smokers *versus* nonsmokers [75]. High TSLP expression was associated with moderate to severe airflow obstruction and heavy smoking in a cohort of Japanese patients with COPD [76].

Epithelial cell-derived TSLP may drive the pathophysiology of COPD *via* effects on a variety of downstream cells. For example, TSLP expression was higher in the airway smooth muscle cells of patients with COPD than in healthy individuals [77]. TSLP can activate airway smooth muscle cell migration and proliferation, which may promote airway remodelling [78].

### IL-25

Less is understood about the role of IL-25 in the pathophysiology of COPD than the other epithelial-derived cytokines. IL-25 promotes inflammation in other respiratory diseases, including severe asthma, in which it induces a T2 inflammatory response through the activation of Th2 cells, basophils, eosinophils and mast cells [7, 79].

Although further research is required to establish a direct role of IL-25 in the pathogenesis of COPD, evidence exists that links IL-25 to some of the cellular changes observed in COPD. For example, goblet and epithelial cell hyperplasia and mucus hypersecretion were induced by IL-25 in mouse models of asthma [80, 81].

Epithelial-derived IL-25 can function in an autocrine manner to induce the expression of IL-25 further, as well as other cytokines such as IL-33 and TSLP [80]. For example, serum IL-25 levels in patients with COPD were significantly higher in those with high TSLP levels than in those with low TSLP levels [74].

## Inflammatory cascade in COPD

The inflammatory phenotypes that occur in COPD are heterogeneous, with some patients experiencing a combination of neutrophilic and eosinophilic inflammation, as demonstrated by sputum analyses of patients with COPD showing mixed inflammatory profiles [5, 82–85]. Comparison of sputum from patients with severe asthma and those with moderate to severe COPD revealed a biological cluster with an overlap of patients with asthma and COPD that was characterised by neutrophilic inflammation (95% of patients with COPD in this cluster had sputum neutrophil predominance) [84]. In contrast, in a distinct cluster comprised mostly of patients with COPD, there was an even balance between sputum neutrophil predominance (49% of patients) and sputum eosinophil predominance (46% of patients), demonstrating the heterogeneity of inflammation in COPD [84]. However, sputum samples only provide information on the proximal airway lumen; therefore, inflammatory profiles may differ in the smaller airway [82, 84]. Adding to the complexity of COPD, the inflammatory phenotype can change over time [85]. Exacerbations also vary between patients with COPD and can be classified according to the underlying degree of inflammation [2]; exacerbation classes include a bacteria-predominant pro-inflammatory endotype, an eosinophil-predominant Th2 endotype, a virus-predominant Th1 endotype and a low inflammatory (pauci-inflammatory) endotype [2].

### Neutrophilic inflammation

Neutrophilic inflammation is characterised by activation of inflammasomes, which are multimeric protein complexes of innate immune system receptors that regulate the activation of caspase-1 and the induction of type 1 and type 3 inflammation [5, 8, 86]. External triggers such as cigarette smoke and oxidants cause airway damage, which leads to the release of pro-inflammatory mediators and damage-associated molecular patterns, including IL-33 and TSLP [5].

CXCL8 release from epithelial cells and lumen macrophages attracts neutrophils *via* CXC receptors 1 and 2 [5, 87]. Granulocyte macrophage-colony stimulating factor is released from airway epithelial cells and regulates macrophage and neutrophil activation, with increased sputum levels seen in patients with COPD compared with healthy individuals [88]. Recruitment of neutrophils can also be induced by epithelial release of IL-17C and subsequent induction of CXCL1, with increased IL-17C release in response to rhinovirus stimulation observed *in vitro* in epithelial cells from patients with COPD compared with healthy individuals [89, 90]. Infiltrating neutrophils produce serine proteases and elastolytic enzymes such as matrix metalloproteinase-8 and -9, and proteinase-3, promoting mucus hypersecretion and small airway fibrosis [91].

Cigarette smoke causes downregulation of ST2 expression in ILC2s and upregulation in macrophages and NK cells, triggering an exaggerated pro-inflammatory cascade through IL-33 [65]. The type 1 inflammatory cascade is also amplified by the impaired structural integrity of the epithelial barrier, which predisposes the airway to invasion of viral and bacterial pathogens, driving a switch of ILC2s to interferon-γ-producing ILC1s [92]. Impaired interferon-γ and interferon-β responses have been observed *in vitro* in airway epithelial cells of smokers compared with nonsmokers [93] and in patients with COPD compared with healthy individuals [94].

The well-balanced microbiome in the upper and lower airways of healthy people can become perturbed in COPD in a process termed dysbiosis [95]. An increase in proteobacteria, including *Haemophilus influenzae*, is observed in some patients with COPD [96, 97]. Such pathogens promote an inflammatory response through the activation of pathogen-associated molecular patterns, triggering the activation of epithelial cells and the release of tumour necrosis factor, IL-1β, IL-6 and IL-8 [5]. Subsequent recruitment of macrophages leads to additional release of pro-inflammatory cytokines, activation of inflammasomes and caspase-1-dependent release of IL-1α, IL-1β, IL-33 and IL-18 [5].

Inflammasome activation can trigger an auto-inflammatory response, leading to intrinsic and persistent production of pro-inflammatory cytokines that is independent of external triggers [98]. Neutrophil recruitment leads to the release of proteases, airway damage and ILC3 activation [5]. Persistent inflammation is driven in part by an autoimmune response. For example, B-cell accumulation is observed in severe COPD, forming tertiary lymphoid follicles alongside T-cells and follicular dendritic cells [5, 99]. The tertiary lymphoid follicles support priming and clonal expansion of B-cells and T-cells [5].

In addition to chronic neutrophilic inflammation, neutrophil extracellular traps (NETs) have been identified in the airways of patients with COPD [100]. A NET component, neutrophil elastase, was shown *in vivo* to impair the epithelial defence against *H. influenzae* infection through the degradation of airway epithelial host defence proteins [100, 101]. In some patients with COPD, IL-6 trans-signalling pathway activation due to chronic colonisation with proteobacteria may be an important driver of disease. In primary human neutrophils, *H. influenzae*-induced NET formation was associated with soluble IL-6 receptor release, and levels of soluble IL-6 receptor were positively correlated with levels of NET surrogate markers in the bronchoalveolar lavage fluid of patients with COPD [102].

### Eosinophilic inflammation

Although less common than neutrophilic inflammation, the prevalence of eosinophilic inflammation in patients with COPD ranges from 10% to 40% [5, 103–105]. Studies assessing sputum cell counts in COPD have reported eosinophil counts ranging from 1.0% to 10.4%, *versus* an average eosinophil count ranging from 0.4% to 1.4% in healthy, nonsmoking individuals [106].

Eosinophilic inflammation is associated with T2 inflammation. Bronchial epithelial and sputum samples from patients with COPD with high eosinophil counts were associated with a genetic profile of T2 inflammation [107, 108]. Eosinophilic inflammation is associated with Th2 cells and ILC2s, which secrete the T2 inflammatory cytokines IL-4, IL-5 and IL-13 in response to epithelial-derived IL-33 and TSLP [5]. IL-5 is required for the survival and maturation of eosinophils, and IL-4 and IL-13 promote activation and trafficking of T2 inflammatory cells and stimulate IgE production from B-cells [5, 8]. The release of chemoattractants, including eotaxin-3, C-C motif chemokine receptor 3 and prostaglandin D2, drives the recruitment of eosinophils to the lung mucosa [5, 8].

IL-4 and IL-5 are also involved downstream in driving and amplifying T2 inflammation in several respiratory diseases, including COPD, whereas IL-13 has unique epithelial-related effects [109]. IL-13 is produced in CD4^+^ T-cells, basophils, eosinophils, mast cells, NK T-cells and ILC2s [109]. ILC2s secrete IL-13 in response to the epithelial-derived cytokines as part of the T2 inflammatory response [8]. IL-13 signals through the IL-4Rα receptor, which is expressed by airway epithelial cells, as well as innate and adaptive immune cells [8].

IL-13 activity is linked to goblet cell hyperplasia and mucus production, as well as smooth muscle contractility and hyperplasia [110–112]. In patients with COPD, bronchial epithelial and sputum gene expression of IL-13 was increased in those with high eosinophil levels *versus* those with low eosinophil levels [113]. Smokers with chronic bronchitis had a higher proportion of IL-13-positive cells in the bronchial submucosa than asymptomatic smokers [114]. Overexpression of IL-13 in the lungs of adult mice resulted in a COPD-like phenotype, characterised by emphysema, enlarged lungs, mucus metaplasia and inflammation [112].

The smoking status of patients with COPD has been shown to affect the inflammatory cascade. Exposure to cigarette smoke can alter gene expression and the release of inflammatory cytokines and chemokines by airway epithelial cells and circulating inflammatory cells [115, 116]. Active smoking has been shown to reduce small airway intraepithelial eosinophil counts in patients with COPD [117].

### Pauci-inflammation

In studies of patients with acute COPD exacerbations, biological clustering analyses have identified patients with a low inflammatory, or pauci-inflammatory, profile [82, 83]. In one study, alongside the subgroups of patients with either eosinophilic (12%), neutrophilic (43%) or mixed profiles (6%), 39% of patients had airway inflammatory infiltrates that were similar to healthy individuals. However, despite having levels of neutrophils and eosinophils within a normal range, these patients had significantly increased levels of inflammatory mediators (sputum IL-6, sputum matrix metalloproteinase-9, serum C-reactive protein and serum amyloid-A) when compared with healthy individuals. The authors speculate that inflammation in nonlung organs may cause airway responses that promote higher levels of inflammatory mediators in the lungs and subsequent development of acute COPD exacerbations [83]. Alternatively, sputum sampling of the upper airway lumen may not fully reflect inflammation within the tissue or lower airways. In comparison, in a second biological clustering study, the proportion of patients with acute COPD exacerbations and a pauci-inflammatory phenotype was only 11%, demonstrating the heterogeneity of inflammation in COPD [82].

## Current and future biomarkers in COPD

Given the complexity of COPD, there is increasing support for the use of biomarkers that enable a more thorough understanding of the variable underlying disease biology in different patients [118]. Biomarkers can be used to guide therapeutic choices, predict individual therapeutic responses to drugs and identify patients who are at risk of poor outcomes [119]. Current and future biomarkers in COPD are summarised here and in table 2.

**TABLE 2:** Current and future biomarkers of COPD

Biomarker	Modality	Use	Limitations
**BEC**	Blood test	Correlate with lung eosinophil counts [8, 104, 120] Predictor of systemic corticosteroid and ICS treatment response [8, 120–122] High BECs associated with FEV_1_ decline [120]	Association between high BECs and FEV_1_ decline is influenced by exacerbation history and prior ICS use [120]
**Sputum eosinophil counts**	Sputum test	Sometimes increased during exacerbations [82] May predict short-term clinical benefit from high-dose ICS [121]	Bacterial infections may suppress sputum eosinophil counts, complicating interpretation [120]
**Periostin**	Blood test	Periostin levels higher in current and former smokers with COPD than in smokers without COPD [123] High periostin levels a potential predictor of ICS and LABA treatment response [124]	Conflicting results for the correlation between periostin levels and ICS responsiveness [123, 124]
**IL-1** **β**	Blood/sputum test	Predictor of bacteria-associated COPD exacerbations [125]	Requires validation in larger, more diverse cohorts of patients [125]
***F*_ENO_**	Exhaled breath sampling	Elevated levels in patients with COPD compared with healthy individuals [126] Levels positively correlate with BECs [8, 127] Predictor of ICS treatment response [128, 129] Potential association with COPD exacerbations [130, 131]	Active smoking may reduce *F*_ENO_ levels [3]
**Transcriptome profiling**	Lung biopsy, bronchial epithelial brushing, peripheral blood	Gene signatures associated with COPD, treatment response and lung function decline [132–135]	The heterogeneity of COPD may limit the use of gene signatures across a broad subset of patients [5, 134]
**Imaging techniques**	CT, magnetic resonance imaging	Emphysema (quantified by CT) as a predictor of lung function decline in smokers with normal lung function [136] Mucus plugs (quantified by CT) as a predictor of treatment response [137]	No standardisation of quantification between CT scanners [138]

BEC: blood eosinophil count; IL: interleukin; *F*_ENO_: fractional exhaled nitric oxide; ICS: inhaled corticosteroid; FEV_1_: forced expiratory volume in 1 s; LABA: long-acting β-agonist; CT: computed tomography.

Blood eosinophil counts (BECs) are elevated in current smokers [139] and in patients with COPD [140], and are a predictive and prognostic COPD biomarker. Many studies, although not all, have observed that BECs correlate with eosinophil counts in the lungs, suggesting that blood samples can be used to assess eosinophilic lung inflammation [8, 104, 120]. Moreover, BECs are predictive of an enhanced response to systemic and inhaled corticosteroids (ICS) and can be used to identify patients who are most likely to benefit from ICS treatment [8, 120–122]. While BECs enable the prediction of ICS therapeutic efficacy in COPD, lung eosinophils do not seem to be the primary target of ICS; instead, high BECs seem to be a biomarker of a broader T2 profile in the lungs that can be modified by ICS treatment [141]. High BECs are also associated with FEV_1_ decline in patients with COPD [142, 143]; however, the relationship between BECs and FEV_1_ decline is influenced by exacerbation history and prior use of ICS [120]. Sputum eosinophil counts are sometimes increased during exacerbations, whereas bacterial infections may suppress sputum eosinophil counts, complicating interpretation [82, 120, 121].

Periostin is an extracellular matrix protein that is secreted by bronchial epithelial cells and is associated with T2 inflammation and ICS responsiveness in asthma [123, 144]. In COPD, periostin levels were higher in current and former smokers with COPD than in smokers without COPD, but did not correlate with T2 inflammation measures or ICS responsiveness [123]. However, another study reported a significant association between high plasma periostin levels and responsiveness to 3-month ICS and long-acting β-agonist (LABA) treatment in patients with stable COPD [124]. Therefore, the utility of periostin as a COPD biomarker remains to be determined.

Several studies have identified IL-1β as a potential biomarker in COPD [145, 146]. Epithelial cell-dependent release of IL-1β and other inflammatory mediators occurs following the detection of pathogens through pathogen-associated molecular patterns [5]. In patients with COPD, sputum IL-1β levels demonstrated utility as a predictive biomarker of bacteria-associated COPD exacerbations [125].

Breathomics (exhaled breath sampling) is a noninvasive method for the detection of disease biomarkers, including fractional exhaled nitric oxide (*F*_ENO_), which is used as a biomarker of T2 inflammation in asthma [3, 8, 147]. A pooled analysis of 24 cross-sectional or case–control studies showed that patients with COPD had mildly elevated *F*_ENO_ levels *versus* heathy individuals and that former smokers with COPD had higher *F*_ENO_ levels than active smokers with COPD [126]. Additionally, *F*_ENO_ levels are positively correlated with BECs in COPD [8, 127]. However, given that active smoking appears to reduce *F*_ENO_ levels, the utility of *F*_ENO_ as a biomarker of T2 inflammation in COPD may be limited [3]. Furthermore, *F*_ENO_ has demonstrated utility as a predictive biomarker for ICS treatment response in COPD [128, 129]. *F*_ENO_ has also been shown to predict dupilumab response in COPD [148]. An association between increased *F*_ENO_ levels and COPD exacerbations has been reported in several studies [130, 131], although an association was not identified in a cohort of Japanese patients with COPD [149].

Transcriptome profiling aims to identify gene signatures from lung biopsies, bronchial epithelial brushings and peripheral blood [132]. Gene signatures have been identified that are associated with COPD, treatment response and lung function decline [132–135]. For example, in patients with COPD, increased activity of a T2 inflammation gene expression signature was found to be associated with eosinophilic inflammation and predicted a favourable response to ICS, while an IL-17 response gene signature was associated with neutrophilic inflammation and a reduced response to ICS [132].

Prognostic imaging biomarkers using techniques such as computed tomography (CT) or magnetic resonance imaging also show promise in COPD. Emphysema, which occurs downstream of epithelial remodelling and destruction, was quantified using CT and predicted a rapid annual decline in FEV_1_ in smokers with normal lung function [136, 150]. Mucus plugs may arise following goblet cell hyperplasia and subsequent mucus hypersecretion in patients with COPD [151]. In CT scans, mucus plugs appear as areas of opacification within the airway lumen and may be quantified using a visual scoring system previously developed for patients with asthma [152]. In the phase 2 FRONTIER-4 study (clinicaltrials.gov identifier NCT04631016) of patients with COPD and chronic bronchitis receiving dual- or triple-inhaled maintenance therapy, the mucus plug score was reduced in patients receiving tozorakimab *versus* placebo, demonstrating the utility of the approach [137]. However, a limitation of CT is the lack of standardisation of quantification between CT scanners, which can make comparisons between images difficult [138].

The current understanding of early COPD biology remains limited. Identification of the early pathophysiological changes that occur in COPD, including the identification of novel epithelial-related biomarkers will be essential for the therapeutic targeting of COPD before the onset of irreversible damage [153]. Supplementary table S1 summarises additional epithelial-derived cytokines and growth factors that are potentially implicated in COPD pathogenesis, and could warrant further investigation as future therapeutic targets.

## Targeting the epithelium and epithelial-derived cytokines to treat COPD

The current standard-of-care treatment approach for COPD involves a combination of bronchodilators (LABAs and long-acting muscarinic antagonists), with ICS use in those at risk of exacerbations [1]. Of note, ICS use should be guided by BECs; a lower likelihood of treatment benefit with ICS in patients with low BECs (<100 cells·µL^−1^) than in those with high BECs (≥100 cells·µL^−1^) has been reported [154]. However, existing therapeutic approaches can fail to prevent COPD exacerbations in certain subgroups of patients, including those with high BECs, and disease burden remains high for many patients with COPD [120, 155–158]. Novel therapeutics that target the inflammatory pathways implicated in COPD, including those that target the epithelial-derived cytokines, are in development for COPD (table 3).

**TABLE 3:** Summary of clinical trial outcomes *versus* placebo for biologics targeting the epithelium and epithelial-derived cytokines in COPD

	Biologic	Study population	Subjects n	Effect on annualised rate of moderate or severe COPD exacerbations *versus* placebo	LS mean difference from baseline *versus* placebo in pre-BD FEV_1_	LS mean difference from baseline *versus* placebo in SGRQ total score
**IL-33**	Itepekimab 300 mg *s.c.* every 2 weeks	Moderate to severe COPD [159]	343	Modified ITT: ↓ 19% Baseline BECs <250 cells·µL^−1^: ↓ 16% Baseline BECs ≥250 cells·µL^−1^: ↓ 22%	Modified ITT: ↑↑ 0.06 L Baseline BECs <250 cells·µL^−1^: ↑ 0.02 L Baseline BECs ≥250 cells·µL^−1^: ↑↑ 0.12 L	NA
	Tozorakimab 600 mg *s.c.* every 4 weeks	Moderate to severe COPD with chronic bronchitis [160]	135	ITT:^#^ ↓ 21% Baseline BECs ≥150 cells·µL^−1^:^#^ NA	ITT: ↑ 0.02 L Baseline BECs ≥150 cells·µL^−1^: ↑ 0.08 L	ITT: NA Baseline BECs ≥150 cells·µL^−1^: NA
**ST2**	Astegolimab 490 mg *s.c.* every 4 weeks	Moderate to very severe COPD [161]	81	ITT: ↓ 22% Baseline BECs ≤170 cells·µL^−1^: ↓ 31% Baseline BECs >170 cells·µL^−1^: ↓ 17% Baseline BECs <300 cells·µL^−1^: ↓ 37% Baseline BECs ≥300 cells·µL^−1^: ↑ 37%	NA	ITT: ↓↓ 3.3 Baseline BECs ≤170 cells·µL^−1^: ↓ 1.3 Baseline BECs >170 cells·µL^−1^: ↓ 5.2 Baseline BECs <300 cells·µL^−1^: NA Baseline BECs ≥300 cells·µL^−1^: NA
**TSLP**	Tezepelumab 420 mg *s.c.* every 4 weeks	Moderate to severe COPD [162]	333	ITT: ↓ 17% Baseline BECs <150 cells·µL^−1^: ↑ 19% Baseline BECs ≥150 to <300 cells·µL^−1^: ↓ 34% Baseline BECs ≥150 cells·µL^−1^: ↓ 37% Baseline BECs ≥300 cells·µL^−1^: ↓ 46%	ITT: ↑ 0.06 L Baseline BECs <150 cells·µL^−1^: ↑ 0.05 L Baseline BECs ≥150 to <300 cells·µL^−1^: ↑ 0.03 L Baseline BECs ≥150 cells·µL^−1^: ↑ 0.06 L Baseline BECs ≥300 cells·µL^−1^: ↑ 0.15 L	ITT: ↓ 2.9 Baseline BECs <150 cells·µL^−1^: ↓ 1.6 Baseline BECs ≥150 to <300 cells·µL^−1^: ↓ 2.4 Baseline BECs ≥150 cells·µL^−1^: ↓ 4.2 Baseline BECs ≥300 cells·µL^−1^: ↓ 9.5
**IL-4R**	Dupilumab 300 mg *s.c.* every 2 weeks	Moderate to severe COPD with chronic bronchitis and baseline BECs ≥300 cells·µL^−1^ [155, 156]	939	ITT: ↓↓ 30% Baseline BECs ≥300 to <500 cells·µL^−1^: ↓ 20% Baseline BECs ≥500 cells·µL^−1^: ↓ 50%	ITT: ↑↑ 0.08 L Baseline BECs ≥300 to <500 cells·µL^−1^: ↑ 0.05 L Baseline BECs ≥500 cells·µL^−1^: ↑ 0.14 L	ITT: ↓↓ 3.4 Baseline BECs ≥300 to <500 cells·µL^−1^: NA Baseline BECs ≥500 cells·µL^−1^: NA
			935	ITT: ↓↓ 34% Baseline BECs <300 cells·µL^−1^: ↓ 38% Baseline BECs ≥300 cells·µL^−1^: ↓ 32%	ITT: ↑↑ 0.06 L Baseline BECs <300 cells·µL^−1^: ↑ 0.03 L Baseline BECs ≥300 cells·µL^−1^: ↑ 0.11 L	ITT: ↓ 3.4 Baseline BECs <300 cells·µL^−1^: NA Baseline BECs ≥300 cells·µL^−1^: NA

↑,↓: a statistically nonsignificant change; ↑↑,↓↓: a statistically significant change; LS: least-squares; BD: bronchodilator; FEV_1_: forced expiratory volume in 1 s; SGRQ: St George's Respiratory Questionnaire; IL: interleukin; ST2: serum stimulated 2; TSLP: thymic stromal lymphopoietin; IL-R: IL receptor; *s.c.*: subcutaneous; ITT: intention-to-treat; BEC: blood eosinophil count; NA: not available. ^#^: time to first COPDCompEx event, a composite end-point that includes worsening of COPD symptoms and moderate or severe exacerbations, which is predictive of efficacy in reducing moderate and severe exacerbations and is suited for small, short-duration, phase 2 studies [160].

Several biologics targeting the IL-33 pathway are being investigated for COPD, including itepekimab, tozorakimab and astegolimab.

Itepekimab is a high-affinity human IgG4 monoclonal antibody that binds and inhibits IL-33, and that has been shown to prevent NF-κB signalling and to block cytokine release from human peripheral blood mononuclear cells [163, 164]. In a phase 2a trial (clinicaltrials.gov identifier NCT03546907) of 343 patients with moderate to severe COPD who were receiving stable dual or triple maintenance therapy, itepekimab did not significantly reduce the rate of COPD exacerbations *versus* placebo (rate ratio 0.81, 95% CI 0.61–1.07; p=0.13) [159]. The treatment effect on acute exacerbations was not associated with baseline BECs. However, improvements in FEV_1_ levels were greater in patients with BECs of ≥250 cells·mm^−3^ compared with those with BECs of <250 cells·mm^−3^ (least-squares mean difference in change from baseline 0.12, 95% CI 0.02–0.21; p=0.016 *versus* 0.02, 95% CI −0.03–0.07; p=0.46). Among current smokers, no reductions in COPD exacerbations or improvements in FEV_1_ levels were observed with itepekimab *versus* placebo. However, the study reported a nominally significant reduction in the rate of exacerbations (rate ratio 0.58, 95% CI 0.39–0.85; p=0.0061) and improved lung function among former smokers with itepekimab *versus* placebo [159]. The efficacy of itepekimab in former smokers with COPD is being investigated in ongoing phase 3 studies (clinicaltrials.gov identifiers NCT04701983 and NCT04751487).

Tozorakimab is a high-affinity human monoclonal antibody with a fast association rate. It inhibits the signalling of both reduced and oxidised IL-33 *via* the ST2 and RAGE-EGFR pathways, respectively [64, 165]. *In vitro*, tozorakimab potently inhibited ST2-dependent inflammatory responses driven by IL-33 in primary human cells and in a mouse model of lung epithelial injury. Furthermore, tozorakimab prevented the oxidation of IL-33 and its activity *via* the RAGE-EGFR signalling pathway, leading to an increase in epithelial cell migration and repair [64, 165]. In the phase 2 FRONTIER-4 study of 135 patients with COPD and chronic bronchitis receiving dual or triple inhaled maintenance therapy, tozorakimab treatment numerically improved pre-bronchodilator FEV_1_ *versus* placebo at week 12. Although this was not statistically significant, greater improvements were seen in patients with two or more exacerbations in the previous 12 months and in those with baseline BECs of ≥150 cells·µL^−1^ [160]. In a time-to-event analysis, tozorakimab numerically reduced the risk of COPDCompEx (a composite outcome measure predictive of a treatment effect on moderate or severe COPD exacerbations [166]) events *versus* placebo at week 28, with greater effect in patients with a higher risk of exacerbations. The effect of tozorakimab on COPDCompEx events was similar in patients with baseline BECs above and below 150 cells·µL^−1^. Treatment effects on lung function and COPDCompEx were similar in current and former smokers [167]. Tozorakimab is currently under investigation in phase 3 studies of patients with COPD with two or more exacerbations in the previous 12 months (clinicaltrials.gov identifiers NCT05166889, NCT05158387, NCT06040086 and NCT05742802).

Astegolimab is a human IgG2 monoclonal antibody that prevents IL-33 from binding to ST2 in solution. Astegolimab binds to the IL-33-binding interface on ST2, directly competing with IL-33 binding, thereby preventing the formation of the active signalling complex necessary for ST2 activation [168]. Treatment with astegolimab did not significantly reduce exacerbation rates *versus* placebo in a phase 2a trial (clinicaltrials.gov identifier NCT03615040) of 81 patients with moderate to very severe COPD (rate ratio, 0.78, 95% CI 0.53–1.14; p=0.19), but did result in significant improvements in health-related quality of life. Patients with BECs of ≤170 cells·µL^−1^ had an exacerbation rate ratio of 0.69 (95% CI 0.39–1.21), whereas those with BECs of >170 cells·µL^−1^ had an exacerbation rate ratio of 0.83 (95% CI 0.49–1.40); a *post hoc* analysis showed that the exacerbation rate ratio for patients receiving astegolimab *versus* placebo favoured those with lower BECs (<300 cells·µL^−1^). This study was not powered to assess efficacy within former and current smoker subgroups, but a *post hoc* analysis showed no differences between these subgroups [161]. In addition, astegolimab is under investigation in patients with COPD who are former or current smokers and who have a history of frequent exacerbations (clinicaltrials.gov identifier NCT05037929).

Tezepelumab is a human monoclonal antibody that binds to TSLP upon its release by epithelial cells, preventing its interaction with the heterodimeric TSLP receptor complex [169]. Tezepelumab therefore blocks TSLP activity and inhibits multiple downstream inflammatory pathways in the airways [162, 169]. In the phase 2a COURSE study (clinicaltrials.gov identifier NCT04039113) of 333 patients with moderate to very severe COPD who had prior exacerbations despite receiving triple inhaled maintenance therapy, a numerical reduction in the annualised rate of moderate or severe COPD exacerbations was observed *versus* placebo (rate ratio 0.83, 90% CI 0.64–1.06; p=0.1042) [162]. Greater reductions were observed in patients with baseline BECs of ≥150 cells·µL^−1^, and smoking history subgroup analysis showed no differences between former and current smokers [162]. Overall, data from COURSE, itepekimab phase 2 and FRONTIER-4 studies showed greater efficacy signals in patients with higher BECs compared with those with lower BECs, although this pattern varied between the clinical end-points studied, and was more consistent across the studies for lung function [159, 162, 167]. In COURSE, greater reductions in exacerbations were observed in patients with higher BECs (*versus* lower BECs) [162], whereas the treatment effect on COPDCompEx events was similar in patients with baseline BECs above and below 150 cells·µL^−1^ in FRONTIER-4 [167]. Adding to this, the astegolimab phase 2a trial demonstrated that the greatest improvements in exacerbation rates were observed in patients with low BECs [161]. While both TSLP and IL-33 are known to regulate T1 and T2 responses [52], blocking the activity of TSLP appears to inhibit more T2-driven effects in COPD, and results with anti-IL-33/ST2 antibodies suggest that the role of IL-33 on exacerbations involves both T2 and non-T2 inflammatory mechanisms.

It is currently unclear whether differential treatment effects will be observed in current and former smokers. Although the phase 2a data with itepekimab have shown greater benefit in former smokers *versus* current smokers [159], the extent to which smoking status influences treatment response varies. The ongoing phase 3 studies AERIFY-1 (clinicaltrials.gov identifier NCT04701983) [170], AERIFY-2 (NCT04751487) [170], ARNASA (NCT05037929) [171], OBERON (NCT05166889) [172], TITANIA (NCT05158387) [173] and MIRANDA (NCT06040086) [174] are expected to provide further clarification.

Biologics that block the T2 cytokines (IL-5, IL-4 and IL-13) and their receptors, which have secondary effects on the epithelium through their actions on these mediators, have been investigated in randomised clinical trials of patients with COPD [155–158, 175, 176]. For example, dupilumab is a human monoclonal antibody that blocks the receptor for IL-4 and IL-13, IL-4Rα. In the phase 3 BOREAS trial (clinicaltrials.gov identifier NCT03930732) of 939 patients with moderate to severe COPD who were receiving triple maintenance therapy (ICS, LABA, long-acting muscarinic antagonist) and had baseline BECs of >300 cells·µL^−1^, dupilumab reduced moderate to severe COPD exacerbations (rate ratio 0.70, 95% CI 0.58–0.86; p<0.001), improved lung function and increased health-related quality of life measures *versus* placebo [155]. Similarly, in the phase 3 NOTUS trial (clinicaltrials.gov identifier NCT04456673) of 721 patients with COPD who had baseline BECs of ≥300 cells·µL^−1^, dupilumab was associated with reduced exacerbations (rate ratio 0.66, 95% CI 0.54–0.82; p<0.001) and improved lung function *versus* placebo [156]. In the phase 3 METREX (clinicaltrials.gov identifier NCT02105948) and METREO (clinicaltrials.gov identifier NCT02105961) studies, the anti-IL-5 monoclonal antibody mepolizumab at a dose of 100 mg was associated with a lower annual rate of moderate or severe exacerbations than placebo among patients with COPD and an eosinophilic phenotype (BECs ≥150 cells·µL^−1^ at screening or ≥300 cells·µL^−1^ at any point in the previous year) [175, 176]. However, in sputum samples from patients with stable COPD, only 20–40% of patients had an eosinophilic phenotype, emphasising an additional need for therapies that reduce exacerbations among the broader population of patients at risk of moderate to severe COPD exacerbations despite receiving triple inhaled therapy.

Icenticaftor, an oral, small-molecule CFTR potentiator, has also been investigated in COPD. In a phase 2 trial (clinicaltrials.gov identifier NCT02449018) of 92 patients with COPD and chronic bronchitis, at day 29, icenticaftor recipients showed no improvement in the change in lung clearance index *versus* placebo recipients, but demonstrated significantly improved lung function and decreased sputum bacterial colonisation [177].

Macrolides, such as azithromycin, are a class of broad-spectrum antibacterials that have been investigated for the treatment of COPD [178]. Macrolides have pleiotropic effects, including inhibition of airway epithelium mucus secretion *in vitro* and *in vivo* [178, 179], mediated in part by inhibition of IL-13-induced MUC5AC expression [180]. Long-term treatment with macrolides has been shown to reduce COPD exacerbations in clinical trials, but concerns remain about the development of antibiotic resistance with sustained macrolide use [181].

The varying clinical trial results underline the complex heterogeneity of COPD. Biologic efficacy could be used in a treatable-traits strategy [182], with the clinical (phenotype) and biological (endotype) characteristics of responder subgroups informing ongoing clinical development and clinical treatment decisions, thereby enabling the delivery of precision medicine approaches in COPD [8].

## Conclusion

COPD is a heterogeneous, complex disease that presents with numerous endotypes and phenotypes. Improving our understanding of the role of the epithelium and epithelial-derived cytokines in driving the inflammatory pathways that are involved in disease progression is essential for the development of targeted therapeutics that will improve COPD treatment outcomes across the different disease phenotypes. Advancing knowledge of the role of the epithelium and epithelial-derived cytokines may clarify our understanding of the factors that drive the development and progression of COPD, allowing earlier identification of those at risk and offering the potential to alter the natural history of the disease.

**Questions for future research**

- Investigation into the role of epithelial-derived cytokines in COPD pathogenesis and into epithelial-related inflammation in COPD.
- Examination of the impact of environmental and patient-related risk factors on epithelial integrity and function.
- Exploration of the genetic factors influencing epithelial responses in COPD.
- Identification and validation of novel biomarkers in COPD, including those related to epithelial function.
- Development of targeted therapies for specific inflammatory phenotypes in COPD.
- Evaluation of the clinical outcomes of interventions targeting epithelial-related inflammation in patients with COPD.

## Supplementary material

**Please note:** supplementary material is not edited by the Editorial Office, and is uploaded as it has been supplied by the author. Supplementary material ERR-0110-2025.SUPPLEMENT

## References

[1] Global Initiative for Chronic Obstructive Lung Disease (GOLD). Global Strategy for the Diagnosis, Management and Prevention of COPD. 2024. Date last accessed: 13 June 2024. https://goldcopd.org/2024-gold-report/

[2] Stolz D, Mkorombindo T, Schumann DM, et al. Towards the elimination of chronic obstructive pulmonary disease: a Lancet Commission. Lancet 2022; 400: 921–972. doi:10.1016/S0140-6736(22)01273-9 36075255 PMC11260396

[3] Flynn C, Brightling C. Is *FeNOtyping* in COPD the path to precision medicine? Respirology 2023; 28: 421–422. doi:10.1111/resp.14474 36811260

[4] Salvi SS, Barnes PJ. Chronic obstructive pulmonary disease in non-smokers. Lancet 2009; 374: 733–743. doi:10.1016/S0140-6736(09)61303-9 19716966

[5] Brightling C, Greening N. Airway inflammation in COPD: progress to precision medicine. Eur Respir J 2019; 54: 1900651. doi:10.1183/13993003.00651-2019 31073084

[6] Lange P, Ahmed E, Lahmar ZM, et al. Natural history and mechanisms of COPD. Respirology 2021; 26: 298–321. doi:10.1111/resp.14007 33506971

[7] Roan F, Obata-Ninomiya K, Ziegler SF. Epithelial cell-derived cytokines: more than just signaling the alarm. J Clin Invest 2019; 129: 1441–1451. doi:10.1172/JCI124606 30932910 PMC6436879

[8] Rabe KF, Rennard S, Martinez FJ, et al. Targeting type 2 inflammation and epithelial alarmins in chronic obstructive pulmonary disease: a biologics outlook. Am J Respir Crit Care Med 2023; 208: 395–405. doi:10.1164/rccm.202303-0455CI 37348121

[9] Aghapour M, Ubags ND, Bruder D, et al. Role of air pollutants in airway epithelial barrier dysfunction in asthma and COPD. Eur Respir Rev 2022; 31: 210112. doi:10.1183/16000617.0112-2021 35321933 PMC9128841

[10] Davies DE. The role of the epithelium in airway remodeling in asthma. Proc Am Thorac Soc 2009; 6: 678–682. doi:10.1513/pats.200907-067DP 20008875 PMC2797070

[11] Russell RJ, Boulet LP, Brightling CE, et al. The airway epithelium: an orchestrator of inflammation, a key structural barrier and a therapeutic target in severe asthma. Eur Respir J 2024; 63: 2301397. doi:10.1183/13993003.01397-2023 38453256 PMC10991852

[12] Hellings PW, Steelant B. Epithelial barriers in allergy and asthma. J Allergy Clin Immunol 2020; 145: 1499–1509. doi:10.1016/j.jaci.2020.04.010 32507228 PMC7270816

[13] Morimoto M, Nishinakamura R, Saga Y, et al. Different assemblies of Notch receptors coordinate the distribution of the major bronchial Clara, ciliated and neuroendocrine cells. Development 2012; 139: 4365–4373. doi:10.1242/dev.083840 23132245 PMC3509731

[14] Rock JR, Gao X, Xue Y, et al. Notch-dependent differentiation of adult airway basal stem cells. Cell Stem Cell 2011; 8: 639–648. doi:10.1016/j.stem.2011.04.003 21624809 PMC3778678

[15] Montoro DT, Haber AL, Biton M, et al. A revised airway epithelial hierarchy includes CFTR-expressing ionocytes. Nature 2018; 560: 319–324. doi:10.1038/s41586-018-0393-7 30069044 PMC6295155

[16] Plasschaert LW, Žilionis R, Choo-Wing R, et al. A single-cell atlas of the airway epithelium reveals the CFTR-rich pulmonary ionocyte. Nature 2018; 560: 377–381. doi:10.1038/s41586-018-0394-6 30069046 PMC6108322

[17] Crystal RG. Airway basal cells. The “smoking gun” of chronic obstructive pulmonary disease. Am J Respir Crit Care Med 2014; 190: 1355–1362. doi:10.1164/rccm.201408-1492PP 25354273 PMC4299651

[18] Raby KL, Michaeloudes C, Tonkin J, et al. Mechanisms of airway epithelial injury and abnormal repair in asthma and COPD. Front Immunol 2023; 14: 1201658. doi:10.3389/fimmu.2023.1201658 37520564 PMC10374037

[19] Bustamante-Marin XM, Ostrowski LE. Cilia and mucociliary clearance. Cold Spring Harb Perspect Biol 2017; 9: a028241. doi:10.1101/cshperspect.a028241 27864314 PMC5378048

[20] Carlier FM, de Fays C, Pilette C. Epithelial barrier dysfunction in chronic respiratory diseases. Front Physiol 2021; 12: 691227. doi:10.3389/fphys.2021.691227 34248677 PMC8264588

[21] Thomas B, Rutman A, Hirst RA, et al. Ciliary dysfunction and ultrastructural abnormalities are features of severe asthma. J Allergy Clin Immunol 2010; 126: 722–729. doi:10.1016/j.jaci.2010.05.046 20673980

[22] Kesimer M. Mucins MUC5AC and MUC5B in the airways: MUCing around together. Am J Respir Crit Care Med 2022; 206: 1055–1057. doi:10.1164/rccm.202208-1459ED 35938865 PMC9704829

[23] Blackburn JB, Li NF, Bartlett NW, et al. An update in club cell biology and its potential relevance to chronic obstructive pulmonary disease. Am J Physiol Lung Cell Mol Physiol 2023; 324: L652–L665. doi:10.1152/ajplung.00192.2022 36942863 PMC10110710

[24] Ontology Lookup Service (OLS). Respiratory tract suprabasal cell. 2024. Date last accessed: 23 September 2024. https://purl.obolibrary.org/obo/CL_4033048

[25] Wu M, Zhang X, Lin Y, et al. Roles of airway basal stem cells in lung homeostasis and regenerative medicine. Respir Res 2022; 23: 122. doi:10.1186/s12931-022-02042-5 35562719 PMC9102684

[26] Cumplido-Laso G, Benitez DA, Mulero-Navarro S, et al. Transcriptional regulation of airway epithelial cell differentiation: insights into the Notch pathway and beyond. Int J Mol Sci 2023; 24: 14789. doi:10.3390/ijms241914789 37834236 PMC10573127

[27] Deprez M, Zaragosi LE, Truchi M, et al. A single-cell atlas of the human healthy airways. Am J Respir Crit Care Med 2020; 202: 1636–1645. doi:10.1164/rccm.201911-2199OC 32726565

[28] Scudieri P, Musante I, Venturini A, et al. Ionocytes and CFTR chloride channel expression in normal and cystic fibrosis nasal and bronchial epithelial cells. Cells 2020; 9: 2090. doi:10.3390/cells9092090 32933106 PMC7565890

[29] Mall MA, Criner GJ, Miravitlles M, et al. Cystic fibrosis transmembrane conductance regulator in COPD: a role in respiratory epithelium and beyond. Eur Respir J 2023; 61: 2201307. doi:10.1183/13993003.01307-2022 37003609 PMC10066568

[30] Romano Ibarra GS, Lei L, Yu W, et al. IL-13 induces loss of CFTR in ionocytes and reduces airway epithelial fluid absorption. J Clin Invest 2024; 134: e181995. doi:10.1172/JCI181995 PMC11527443 39255033

[31] Gu X, Karp PH, Brody SL, et al. Chemosensory functions for pulmonary neuroendocrine cells. Am J Respir Cell Mol Biol 2014; 50: 637–646. doi:10.1165/rcmb.2013-0199OC 24134460 PMC4068934

[32] Seeholzer LF, Julius D. Neuroendocrine cells initiate protective upper airway reflexes. Science 2024; 384: 295–301. doi:10.1126/science.adh5483 38669574 PMC11407116

[33] Candeli N, Dayton T. Investigating pulmonary neuroendocrine cells in human respiratory diseases with airway models. Dis Model Mech 2024; 17: dmm050620. doi:10.1242/dmm.050620 38813849 PMC11152561

[34] Hollenhorst MI, Krasteva-Christ G. Chemosensory cells in the respiratory tract as crucial regulators of innate immune responses. J Physiol 2023; 601: 1555–1572. doi:10.1113/JP282307 37009787

[35] Perniss A, Liu S, Boonen B, et al. Chemosensory cell-derived acetylcholine drives tracheal mucociliary clearance in response to virulence-associated formyl peptides. Immunity 2020; 52: 683–699.e11. doi:10.1016/j.immuni.2020.03.005 32294408

[36] Hammad H, Chieppa M, Perros F, et al. House dust mite allergen induces asthma *via* Toll-like receptor 4 triggering of airway structural cells. Nat Med 2009; 15: 410–416. doi:10.1038/nm.1946 19330007 PMC2789255

[37] Crystal RG, Randell SH, Engelhardt JF, et al. Airway epithelial cells: current concepts and challenges. Proc Am Thorac Soc 2008; 5: 772–777. doi:10.1513/pats.200805-041HR 18757316 PMC5820806

[38] Hogg JC, McDonough JE, Suzuki M. Small airway obstruction in COPD: new insights based on micro-CT imaging and MRI imaging. Chest 2013; 143: 1436–1443. doi:10.1378/chest.12-1766 23648907 PMC3653349

[39] Thomas B, Koh MS, O'Callaghan C, et al. Dysfunctional bronchial cilia are a feature of chronic obstructive pulmonary disease (COPD). COPD 2021; 18: 657–663. doi:10.1080/15412555.2021.1963695 34468237

[40] Staudt MR, Buro-Auriemma LJ, Walters MS, et al. Airway basal stem/progenitor cells have diminished capacity to regenerate airway epithelium in chronic obstructive pulmonary disease. Am J Respir Crit Care Med 2014; 190: 955–958. doi:10.1164/rccm.201406-1167LE 25317467 PMC4299582

[41] Yaghi A, Zaman A, Cox G, et al. Ciliary beating is depressed in nasal cilia from chronic obstructive pulmonary disease subjects. Respir Med 2012; 106: 1139–1147. doi:10.1016/j.rmed.2012.04.001 22608352

[42] Rogers DF. The airway goblet cell. Int J Biochem Cell Biol 2003; 35: 1–6. doi:10.1016/S1357-2725(02)00083-3 12467641

[43] Caramori G, Di Gregorio C, Carlstedt I, et al. Mucin expression in peripheral airways of patients with chronic obstructive pulmonary disease. Histopathology 2004; 45: 477–484. doi:10.1111/j.1365-2559.2004.01952.x 15500651

[44] Kotlyarov S. Involvement of the innate immune system in the pathogenesis of chronic obstructive pulmonary disease. Int J Mol Sci 2022; 23: 985. doi:10.3390/ijms23020985 35055174 PMC8778852

[45] McDonough JE, Yuan R, Suzuki M, et al. Small-airway obstruction and emphysema in chronic obstructive pulmonary disease. N Engl J Med 2011; 365: 1567–1575. doi:10.1056/NEJMoa1106955 22029978 PMC3238466

[46] Singh D. Small airway disease in patients with chronic obstructive pulmonary disease. Tuberc Respir Dis 2017; 80: 317–324. doi:10.4046/trd.2017.0080 PMC5617847 28905527

[47] Heijink IH, Noordhoek JA, Timens W, et al. Abnormalities in airway epithelial junction formation in chronic obstructive pulmonary disease. Am J Respir Crit Care Med 2014; 189: 1439–1442. doi:10.1164/rccm.201311-1982LE 24881942

[48] Hackett NR, Shaykhiev R, Walters MS, et al. The human airway epithelial basal cell transcriptome. PLoS One 2011; 6: e18378. doi:10.1371/journal.pone.0018378 21572528 PMC3087716

[49] Okuda K, Dang H, Kobayashi Y, et al. Secretory cells dominate airway CFTR expression and function in human airway superficial epithelia. Am J Respir Crit Care Med 2021; 203: 1275–1289. doi:10.1164/rccm.202008-3198OC 33321047 PMC8456462

[50] Lin C-R, Bahmed K, Kosmider B. Dysregulated cell signaling in pulmonary emphysema. Front Med 2022; 8: 762878. doi:10.3389/fmed.2021.762878 PMC8762198 35047522

[51] Hadzic S, Wu C-Y, Avdeev S, et al. Lung epithelium damage in COPD – an unstoppable pathological event? Cell Signal 2020; 68: 109540. doi:10.1016/j.cellsig.2020.109540 31953012

[52] Calderon AA, Dimond C, Choy DF, et al. Targeting interleukin-33 and thymic stromal lymphopoietin pathways for novel pulmonary therapeutics in asthma and COPD. Eur Respir Rev 2023; 32: 220144. doi:10.1183/16000617.0144-2022 36697211 PMC9879340

[53] Varricchi G, Poto R. Towards precision medicine in COPD: targeting type 2 cytokines and alarmins. Eur J Intern Med 2024; 125: 28–31. doi:10.1016/j.ejim.2024.05.011 38762432

[54] Furci F, Murdaca G, Pelaia C, et al. TSLP and HMGB1: inflammatory targets and potential biomarkers for precision medicine in asthma and COPD. Biomedicines 2023; 11: 437. doi:10.3390/biomedicines11020437 36830972 PMC9953666

[55] Duchesne M, Okoye I, Lacy P. Epithelial cell alarmin cytokines: frontline mediators of the asthma inflammatory response. Front Immunol 2022; 13: 975914. doi:10.3389/fimmu.2022.975914 36311787 PMC9616080

[56] Katz-Kiriakos E, Steinberg DF, Kluender CE, et al. Epithelial IL-33 appropriates exosome trafficking for secretion in chronic airway disease. JCI Insight 2021; 6: e136166. 33507882 10.1172/jci.insight.136166 PMC7934940

[57] Bernard O, Lachowicz-Scroggins M, Sharp L, et al. Pyroptosis is a novel mechanism of IL-33 release from airway epithelial cells. Am J Respir Crit Care Med 2018; 197: A7799.

[58] Cayrol C, Girard JP. Interleukin-33 (IL-33): a nuclear cytokine from the IL-1 family. Immunol Rev 2018; 281: 154–168. doi:10.1111/imr.12619 29247993

[59] Chen W, Chen S, Yan C, et al. Allergen protease-activated stress granule assembly and gasdermin D fragmentation control interleukin-33 secretion. Nat Immunol 2022; 23: 1021–1030. doi:10.1038/s41590-022-01255-6 35794369 PMC11345751

[60] Drake LY, Kita H. IL-33: biological properties, functions, and roles in airway disease. Immunol Rev 2017; 278: 173–184. doi:10.1111/imr.12552 28658560 PMC5492954

[61] Afferni C, Buccione C, Andreone S, et al. The pleiotropic immunomodulatory functions of IL-33 and its implications in tumor immunity. Front Immunol 2018; 9: 2601. doi:10.3389/fimmu.2018.02601 30483263 PMC6242976

[62] Poto R, Gambardella AR, Marone G, et al. Basophils from allergy to cancer. Front Immunol 2022; 13: 1056838. doi:10.3389/fimmu.2022.1056838 36578500 PMC9791102

[63] Cohen ES, Scott IC, Majithiya JB, et al. Oxidation of the alarmin IL-33 regulates ST2-dependent inflammation. Nat Commun 2015; 6: 8327. doi:10.1038/ncomms9327 26365875 PMC4579851

[64] Strickson S, Houslay KF, Negri VA, et al. Oxidised IL-33 drives COPD epithelial pathogenesis *via* ST2-independent RAGE/EGFR signalling complex. Eur Respir J 2023; 62: 2202210. doi:10.1183/13993003.02210-2022 37442582 PMC10533947

[65] Kearley J, Silver JS, Sanden C, et al. Cigarette smoke silences innate lymphoid cell function and facilitates an exacerbated type I interleukin-33-dependent response to infection. Immunity 2015; 42: 566–579. doi:10.1016/j.immuni.2015.02.011 25786179

[66] Byers DE, Alexander-Brett J, Patel AC, et al. Long-term IL-33-producing epithelial progenitor cells in chronic obstructive lung disease. J Clin Invest 2013; 123: 3967–3982. doi:10.1172/JCI65570 23945235 PMC3754239

[67] Joo H, Park SJ, Min KH, et al. Association between plasma interleukin-33 level and acute exacerbation of chronic obstructive pulmonary disease. BMC Pulm Med 2021; 21: 86. doi:10.1186/s12890-021-01423-8 33722239 PMC7962403

[68] Abdo M, Pedersen F, Kirsten AM, et al. Association of airway inflammation and smoking status with IL-33 level in sputum of patients with asthma or COPD. Eur Respir J 2024; 64: 2400347. doi:10.1183/13993003.00347-2024 39147409 PMC11424925

[69] Faiz A, Mahbub RM, Boedijono FS, et al. IL-33 expression is lower in current smokers at both transcriptomic and protein levels. Am J Respir Crit Care Med 2023; 208: 1075–1087. doi:10.1164/rccm.202210-1881OC 37708400 PMC10867944

[70] Tworek D, Majewski S, Szewczyk K, et al. The association between airway eosinophilic inflammation and IL-33 in stable non-atopic COPD. Respir Res 2018; 19: 108. doi:10.1186/s12931-018-0807-y 29859068 PMC5984757

[71] Anzalone G, Albano GD, Montalbano AM, et al. IL-17A-associated IKK-α signaling induced TSLP production in epithelial cells of COPD patients. Exp Mol Med 2018; 50: 1–12. doi:10.1038/s12276-018-0158-2 PMC6173689 30291224

[72] Ziegler SF, Roan F, Bell BD, et al. The biology of thymic stromal lymphopoietin (TSLP). Adv Pharmacol 2013; 66: 129–155. doi:10.1016/B978-0-12-404717-4.00004-4 23433457 PMC4169878

[73] Ying S, O'Connor B, Ratoff J, et al. Expression and cellular provenance of thymic stromal lymphopoietin and chemokines in patients with severe asthma and chronic obstructive pulmonary disease. J Immunol 2008; 181: 2790–2798. doi:10.4049/jimmunol.181.4.2790 18684970

[74] Wu L, Fang L, Xu X, et al. Effect of TSLP on the function of platelets and IL-25 in chronic obstructive pulmonary disease. Int J Clin Exp Med 2019; 12: 4942–4948.

[75] Paplinska-Goryca M, Misiukiewicz-Stepien P, Nejman-Gryz P, et al. Epithelial-macrophage-dendritic cell interactions impact alarmins expression in asthma and COPD. Clinical Immunology 2020; 215: 108421. doi:10.1016/j.clim.2020.108421 32302698

[76] Yamada H, Hida N, Masuko H, et al. Effects of lung function-related genes and *TSLP* on COPD phenotypes. COPD 2020; 17: 59–64. doi:10.1080/15412555.2019.1708296 31910693

[77] Zhang K, Shan L, Rahman MS, et al. Constitutive and inducible thymic stromal lymphopoietin expression in human airway smooth muscle cells: role in chronic obstructive pulmonary disease. Am J Physiol Lung Cell Mol Physiol 2007; 293: L375–L382. doi:10.1152/ajplung.00045.2007 17513456

[78] Redhu NS, Shan L, Movassagh H, et al. Thymic stromal lymphopoietin induces migration in human airway smooth muscle cells. Sci Rep 2013; 3: 2301. doi:10.1038/srep02301 23892442 PMC3725475

[79] Chan R, Stewart K, Misirovs R, et al. Targeting downstream type 2 cytokines or upstream epithelial alarmins for severe asthma. J Allergy Clin Immunol Pract 2022; 10: 1497–1505. doi:10.1016/j.jaip.2022.01.040 35131510

[80] Borowczyk J, Shutova M, Brembilla NC, et al. IL-25 (IL-17E) in epithelial immunology and pathophysiology. J Allergy Clin Immunol 2021; 148: 40–52. doi:10.1016/j.jaci.2020.12.628 33485651

[81] Angkasekwinai P, Park H, Wang YH, et al. Interleukin 25 promotes the initiation of proallergic type 2 responses. J Exp Med 2007; 204: 1509–1517. doi:10.1084/jem.20061675 17562814 PMC2118650

[82] Bafadhel M, McKenna S, Terry S, et al. Acute exacerbations of chronic obstructive pulmonary disease: identification of biologic clusters and their biomarkers. Am J Respir Crit Care Med 2011; 184: 662–671. doi:10.1164/rccm.201104-0597OC 21680942

[83] Gao P, Zhang J, He X, et al. Sputum inflammatory cell-based classification of patients with acute exacerbation of chronic obstructive pulmonary disease. PLoS One 2013; 8: e57678. doi:10.1371/journal.pone.0057678 23741289 PMC3669375

[84] Ghebre MA, Bafadhel M, Desai D, et al. Biological clustering supports both “Dutch” and “British” hypotheses of asthma and chronic obstructive pulmonary disease. J Allergy Clin Immunol 2015; 135: 63–72. doi:10.1016/j.jaci.2014.06.035 25129678 PMC4282726

[85] David B, Bafadhel M, Koenderman L, et al. Eosinophilic inflammation in COPD: from an inflammatory marker to a treatable trait. Thorax 2021; 76: 188–195. doi:10.1136/thoraxjnl-2020-215167 33122447 PMC7815887

[86] Guo H, Callaway JB, Ting JPY. Inflammasomes: mechanism of action, role in disease, and therapeutics. Nat Med 2015; 21: 677–687. doi:10.1038/nm.3893 26121197 PMC4519035

[87] da Silva CO, Gicquel T, Daniel Y, et al. Alteration of immunophenotype of human macrophages and monocytes after exposure to cigarette smoke. Sci Rep 2020; 10: 12796. doi:10.1038/s41598-020-68753-1 32732964 PMC7393094

[88] Saha S, Doe C, Mistry V, et al. Granulocyte–macrophage colony-stimulating factor expression in induced sputum and bronchial mucosa in asthma and COPD. Thorax 2009; 64: 671–676. doi:10.1136/thx.2008.108290 19213775 PMC2712140

[89] Jamieson KC, Traves SL, Kooi C, et al. Rhinovirus and bacteria synergistically induce IL-17C release from human airway epithelial cells to promote neutrophil recruitment. J Immunol 2019; 202: 160–170. doi:10.4049/jimmunol.1800547 30504421

[90] Jamieson KC, Wiehler S, Michi AN, et al. Rhinovirus induces basolateral release of IL-17C in highly differentiated airway epithelial cells. Front Cell Infect Microbiol 2020; 10: 103. doi:10.3389/fcimb.2020.00103 32232015 PMC7082745

[91] Butler A, Walton GM, Sapey E. Neutrophilic inflammation in the pathogenesis of chronic obstructive pulmonary disease. COPD 2018; 15: 392–404. doi:10.1080/15412555.2018.1476475 30064276

[92] Silver JS, Kearley J, Copenhaver AM, et al. Inflammatory triggers associated with exacerbations of COPD orchestrate plasticity of group 2 innate lymphoid cells in the lungs. Nat Immunol 2016; 17: 626–635. doi:10.1038/ni.3443 27111143 PMC5345745

[93] Wu W, Zhang W, Booth JL, et al. Human primary airway epithelial cells isolated from active smokers have epigenetically impaired antiviral responses. Respir Res 2016; 17: 111. doi:10.1186/s12931-016-0428-2 27604339 PMC5013564

[94] Veerati PC, Troy NM, Reid AT, et al. Airway epithelial cell immunity is delayed during rhinovirus infection in asthma and COPD. Front Immunol 2020; 11: 974. doi:10.3389/fimmu.2020.00974 32499788 PMC7243842

[95] Sin DD. Chronic obstructive pulmonary disease and the airway microbiome: what respirologists need to know. Tuberc Respir Dis 2023; 86: 166–175. doi:10.4046/trd.2023.0015 PMC10323206 37038880

[96] Bafadhel M, Haldar K, Barker B, et al. Airway bacteria measured by quantitative polymerase chain reaction and culture in patients with stable COPD: relationship with neutrophilic airway inflammation, exacerbation frequency, and lung function. Int J Chron Obstruct Pulmon Dis 2015; 10: 1075–1083. doi:10.2147/COPD.S80091 26089657 PMC4468933

[97] Wang Z, Singh R, Miller BE, et al. Sputum microbiome temporal variability and dysbiosis in chronic obstructive pulmonary disease exacerbations: an analysis of the COPDMAP study. Thorax 2018; 73: 331–338. doi:10.1136/thoraxjnl-2017-210741 29269441

[98] Scambler T, Holbrook J, Savic S, et al. Autoinflammatory disease in the lung. Immunology 2018; 154: 563–573. doi:10.1111/imm.12937 29676014 PMC6050210

[99] Hogg JC, Chu F, Utokaparch S, et al. The nature of small-airway obstruction in chronic obstructive pulmonary disease. N Engl J Med 2004; 350: 2645–2653. doi:10.1056/NEJMoa032158 15215480

[100] Keir HR, Chalmers JD. Neutrophil extracellular traps in chronic lung disease: implications for pathogenesis and therapy. Eur Respir Rev 2022; 31: 210241. doi:10.1183/16000617.0241-2021 35197267 PMC9488971

[101] Jiang D, Wenzel SE, Wu Q, et al. Human neutrophil elastase degrades SPLUNC1 and impairs airway epithelial defense against bacteria. PLoS One 2013; 8: e64689. doi:10.1371/journal.pone.0064689 23741370 PMC3669426

[102] Winslow S, Odqvist L, Diver S, et al. Multi-omics links IL-6 trans-signalling with neutrophil extracellular trap formation and *Haemophilus* infection in COPD. Eur Respir J 2021; 58: 2003312. doi:10.1183/13993003.03312-2020 33766947

[103] George L, Brightling CE. Eosinophilic airway inflammation: role in asthma and chronic obstructive pulmonary disease. Ther Adv Chronic Dis 2016; 7: 34–51. doi:10.1177/2040622315609251 26770668 PMC4707428

[104] Singh D, Kolsum U, Brightling CE, et al. Eosinophilic inflammation in COPD: prevalence and clinical characteristics. Eur Respir J 2014; 44: 1697–1700. doi:10.1183/09031936.00162414 25323230

[105] Yun JH, Lamb A, Chase R, et al. Blood eosinophil count thresholds and exacerbations in patients with chronic obstructive pulmonary disease. J Allergy Clin Immunol 2018; 141: 2037–2047.e10. doi:10.1016/j.jaci.2018.04.010 29709670 PMC5994197

[106] Higham A, Beech A, Singh D. The relevance of eosinophils in chronic obstructive pulmonary disease: inflammation, microbiome, and clinical outcomes. J Leukoc Biol 2024; 116: 927–946. doi: 10.1093/jleuko/qiae153 38941350

[107] Higham A, Beech A, Wolosianka S, et al. Type 2 inflammation in eosinophilic chronic obstructive pulmonary disease. Allergy 2021; 76: 1861–1864. doi:10.1111/all.14661 33206402 PMC8247000

[108] Kolsum U, Damera G, Pham TH, et al. Pulmonary inflammation in patients with chronic obstructive pulmonary disease with higher blood eosinophil counts. J Allergy Clin Immunol 2017; 140: 1181–1184.e7. doi:10.1016/j.jaci.2017.04.027 28506852

[109] Junttila IS. Tuning the cytokine responses: an update on interleukin (IL)-4 and IL-13 receptor complexes. Front Immunol 2018; 9: 888. doi:10.3389/fimmu.2018.00888 29930549 PMC6001902

[110] Gandhi NA, Bennett BL, Graham NMH, et al. Targeting key proximal drivers of type 2 inflammation in disease. Nat Rev Drug Discov 2016; 15: 35–50. doi:10.1038/nrd4624 26471366

[111] Fahy JV. Type 2 inflammation in asthma – present in most, absent in many. Nat Rev Immunol 2015; 15: 57–65. doi:10.1038/nri3786 25534623 PMC4390063

[112] Zheng T, Zhu Z, Wang Z, et al. Inducible targeting of IL-13 to the adult lung causes matrix metalloproteinase- and cathepsin-dependent emphysema. J Clin Invest 2000; 106: 1081–1093. doi:10.1172/JCI10458 11067861 PMC301418

[113] Lee JS, Rosengart MR, Kondragunta V, et al. Inverse association of plasma IL-13 and inflammatory chemokines with lung function impairment in stable COPD: a cross-sectional cohort study. Respir Res 2007; 8: 64. doi:10.1186/1465-9921-8-64 17868461 PMC2064925

[114] Miotto D, Ruggieri MP, Boschetto P, et al. Interleukin-13 and -4 expression in the central airways of smokers with chronic bronchitis. Eur Respir J 2003; 22: 602–608. doi:10.1183/09031936.03.00046402 14582911

[115] Chung KF. Cytokines in chronic obstructive pulmonary disease. Eur Respir J 2001; 18: Suppl. 34, 50s–59s. doi:10.1183/09031936.01.00229701 12392035

[116] Mulvanny A, Pattwell C, Beech A, et al. Validation of sputum biomarker immunoassays and cytokine expression profiles in COPD. Biomedicines 2022; 10: 1949. doi:10.3390/biomedicines10081949 36009496 PMC9405928

[117] Beech A, Booth S, Higham A, et al. Current smoking reduces small airway eosinophil counts in COPD. ERJ Open Res 2024; 10: 00870-2023. doi:10.1183/23120541.00870-2023 38259811 PMC10801758

[118] Sidhaye VK, Nishida K, Martinez FJ. Precision medicine in COPD: where are we and where do we need to go? Eur Respir Rev 2018; 27: 180022. doi:10.1183/16000617.0022-2018 30068688 PMC6156790

[119] Leung JM, Obeidat M, Sadatsafavi M, et al. Introduction to precision medicine in COPD. Eur Respir J 2019; 53: 1802460. doi:10.1183/13993003.02460-2018 30679189

[120] Singh D, Agusti A, Martinez FJ, et al. Blood eosinophils and chronic obstructive pulmonary disease: a Global Initiative for Chronic Obstructive Lung Disease Science Committee 2022 review. Am J Respir Crit Care Med 2022; 206: 17–24. doi:10.1164/rccm.202201-0209PP 35737975

[121] Leigh R, Pizzichini MMM, Morris MM, et al. Stable COPD: predicting benefit from high-dose inhaled corticosteroid treatment. Eur Respir J 2006; 27: 964–971. doi:10.1183/09031936.06.00072105 16446316

[122] Singh D, Bafadhel M, Brightling CE, et al. Blood eosinophil counts in clinical trials for chronic obstructive pulmonary disease. Am J Respir Crit Care Med 2020; 202: 660–671. doi:10.1164/rccm.201912-2384PP 32186896 PMC7462391

[123] Carpaij OA, Muntinghe FOW, Wagenaar MB, et al. Serum periostin does not reflect type 2-driven inflammation in COPD. Respir Res 2018; 19: 112. doi:10.1186/s12931-018-0818-8 29879994 PMC5992772

[124] Park HY, Lee H, Koh WJ, et al. Association of blood eosinophils and plasma periostin with FEV_1_ response after 3-month inhaled corticosteroid and long-acting beta_2_-agonist treatment in stable COPD patients. Int J Chron Obstruct Pulmon Dis 2015; 11: 23–30. doi: 10.2147/COPD.S94797 26730185 PMC4694663

[125] Budroni S, Taccone M, Stella M, et al. Cytokine biomarkers of exacerbations in sputum from chronic obstructive pulmonary disease patients: a prospective cohort study. J Infect Dis 2024; 230: e1112–e1120. doi:10.1093/infdis/jiae232 38836471 PMC11566228

[126] Lu Z, Huang W, Wang L, et al. Exhaled nitric oxide in patients with chronic obstructive pulmonary disease: a systematic review and meta-analysis. Int J Chron Obstruct Pulmon Dis 2018; 13: 2695–2705. doi:10.2147/COPD.S165780 30214187 PMC6124452

[127] Vincken S, Sylvia V, Daniel S, et al. The role of *F*_ENO_ in stable COPD patients with eosinophilic airway inflammation. Respir Med 2021; 181: 106377. doi:10.1016/j.rmed.2021.106377 33838525

[128] Yamaji Y, Oishi K, Hamada K, et al. Detection of type2 biomarkers for response in COPD. J Breath Res 2020; 14: 026007. doi:10.1088/1752-7163/ab71a4 32000146

[129] Wu YK, Su WL, Huang CY, et al. Treatment of chronic obstructive pulmonary disease in patients with different fractional exhaled nitric oxide levels. Medicine 2018; 97: e11922. doi:10.1097/MD.0000000000011922 30461600 PMC6392665

[130] Alcázar-Navarrete B, Ruiz Rodríguez O, Conde Baena P, et al. Persistently elevated exhaled nitric oxide fraction is associated with increased risk of exacerbation in COPD. Eur Respir J 2018; 51: 1701457. doi:10.1183/13993003.01457-2017 29348180

[131] Liu X, Zhang H, Wang Y, et al. Fractional exhaled nitric oxide is associated with the severity of stable COPD. COPD 2020; 17: 121–127. doi:10.1080/15412555.2019.1704231 32116037

[132] van den Berge M, Faiz A. Transcriptome-based signatures: the future biomarkers in obstructive pulmonary diseases such as asthma and chronic obstructive pulmonary disease? Am J Respir Crit Care Med 2022; 205: 139–140. doi:10.1164/rccm.202110-2353ED 34793289 PMC8787239

[133] Becker EJ, Faiz A, van den Berge M, et al. Bronchial gene expression signature associated with rate of subsequent FEV_1_ decline in individuals with and at risk of COPD. Thorax 2022; 77: 31–39. doi:10.1136/thoraxjnl-2019-214476 33972452 PMC13021120

[134] Steiling K, van den Berge M, Hijazi K, et al. A dynamic bronchial airway gene expression signature of chronic obstructive pulmonary disease and lung function impairment. Am J Respir Crit Care Med 2013; 187: 933–942. doi:10.1164/rccm.201208-1449OC 23471465 PMC3707363

[135] van den Berge M, Steiling K, Timens W, et al. Airway gene expression in COPD is dynamic with inhaled corticosteroid treatment and reflects biological pathways associated with disease activity. Thorax 2014; 69: 14–23. doi:10.1136/thoraxjnl-2012-202878 23925644 PMC3888587

[136] Yuan R, Hogg JC, Paré PD, et al. Prediction of the rate of decline in FEV_1_ in smokers using quantitative computed tomography. Thorax 2009; 64: 944–949. doi:10.1136/thx.2008.112433 19734130 PMC3035577

[137] Nordenmark L, Guller P, Reid F, et al. S91 Tozorakimab (anti-IL-33 mAb) reduces mucus plugging in COPD: an imaging sub-study in the FRONTIER-4 phase 2a COPD trial. Thorax 2024; 79: A67–A68.

[138] Washko GR. Diagnostic imaging in COPD. Semin Respir Crit Care Med 2010; 31: 276–285. doi:10.1055/s-0030-1254068 20496297 PMC4334134

[139] Benson VS, Hartl S, Barnes N, et al. Blood eosinophil counts in the general population and airways disease: a comprehensive review and meta-analysis. Eur Respir J 2022; 59: 2004590. doi:10.1183/13993003.04590-2020 34172466 PMC8756293

[140] Kolsum U, Southworth T, Jackson N, et al. Blood eosinophil counts in COPD patients compared to controls. Eur Respir J 2019; 54: 1900633. doi:10.1183/13993003.00633-2019 31221811

[141] Lea S, Higham A, Beech A, et al. How inhaled corticosteroids target inflammation in COPD. Eur Respir Rev 2023; 32: 230084. doi:10.1183/16000617.0084-2023 37852657 PMC10582931

[142] Hong YS, Park HY, Ryu S, et al. The association of blood eosinophil counts and FEV_1_ decline: a cohort study. Eur Respir J 2024; 63: 2301037. doi:10.1183/13993003.01037-2023 38636990

[143] Tan WC, Bourbeau J, Nadeau G, et al. High eosinophil counts predict decline in FEV_1_: results from the CanCOLD study. Eur Respir J 2021; 57: 2000838. doi:10.1183/13993003.00838-2020 33303555

[144] Nejman-Gryz P, Górska K, Paplińska-Goryca M, et al. Periostin and thymic stromal lymphopoietin-potential crosstalk in obstructive airway diseases. J Clin Med 2020; 9: 3667. doi:10.3390/jcm9113667 33203095 PMC7696351

[145] Zou Y, Chen X, Liu J, et al. Serum IL-1β and IL-17 levels in patients with COPD: associations with clinical parameters. Int J Chron Obstruct Pulmon Dis 2017; 12: 1247–1254. doi:10.2147/COPD.S131877 28490868 PMC5413485

[146] Singh S, Verma SK, Kumar S, et al. Correlation of severity of chronic obstructive pulmonary disease with potential biomarkers. Immunol Lett 2018; 196: 1–10. doi:10.1016/j.imlet.2018.01.004 29329680

[147] Kiss H, Örlős Z, Gellért Á, et al. Exhaled biomarkers for point-of-care diagnosis: recent advances and new challenges in breathomics. Micromachines 2023; 14: 391. doi:10.3390/mi14020391 36838091 PMC9964519

[148] Christenson S, Hanania NA, Bhatt SP, et al. In the phase 3 Boreas trial, baseline blood eosinophils and baseline fractional exhaled nitric oxide levels predict the response to dupilumab in patients with moderate-to-severe chronic obstructive pulmonary disease and type 2 inflammation. Am J Respir Crit Care Med 2024; 209: A6620.

[149] Kobayashi S, Hanagama M, Ishida M, et al. Exhaled nitric oxide: a biomarker for chronic obstructive pulmonary disease. Respir Investig 2021; 59: 364–366. doi:10.1016/j.resinv.2021.01.003 33602651

[150] Hogg JC, Pare PD, Hackett TL. The contribution of small airway obstruction to the pathogenesis of chronic obstructive pulmonary disease. Physiol Rev 2017; 97: 529–552. doi:10.1152/physrev.00025.2015 28151425 PMC6151481

[151] Shaykhiev R. Emerging biology of persistent mucous cell hyperplasia in COPD. Thorax 2019; 74: 4–6. doi:10.1136/thoraxjnl-2018-212271 30266881 PMC6347109

[152] Dunican EM, Elicker BM, Gierada DS, et al. Mucus plugs in patients with asthma linked to eosinophilia and airflow obstruction. J Clin Invest 2018; 128: 997–1009. doi:10.1172/JCI95693 29400693 PMC5824874

[153] Soriano JB, Polverino F, Cosio BG. What is early COPD and why is it important? Eur Respir J 2018; 52: 1801448. doi:10.1183/13993003.01448-2018 30309976

[154] Ashdown HF, Smith M, McFadden E, et al. Blood eosinophils to guide inhaled maintenance therapy in a primary care COPD population. ERJ Open Res 2021; 8: 00606-2021. doi:10.1183/23120541.00606-2021 35141324 PMC8819252

[155] Bhatt SP, Rabe KF, Hanania NA, et al. Dupilumab for COPD with type 2 inflammation indicated by eosinophil counts. N Engl J Med 2023; 389: 205–214. doi:10.1056/NEJMoa2303951 37272521

[156] Bhatt SP, Rabe KF, Hanania NA, et al. Dupilumab for COPD with blood eosinophil evidence of type 2 inflammation. N Engl J Med 2024; 390: 2274–2283. doi:10.1056/NEJMoa2401304 38767614

[157] Criner GJ, Celli BR, Brightling CE, et al. Benralizumab for the prevention of COPD exacerbations. N Engl J Med 2019; 381: 1023–1034. doi:10.1056/NEJMoa1905248 31112385

[158] Criner GJ, Celli BR, Singh D, et al. Predicting response to benralizumab in chronic obstructive pulmonary disease: analyses of GALATHEA and TERRANOVA studies. Lancet Respir Med 2020; 8: 158–170. doi:10.1016/S2213-2600(19)30338-8 31575508

[159] Rabe KF, Celli BR, Wechsler ME, et al. Safety and efficacy of itepekimab in patients with moderate-to-severe COPD: a genetic association study and randomised, double-blind, phase 2a trial. Lancet Respir Med 2021; 9: 1288–1298. doi:10.1016/S2213-2600(21)00167-3 34302758

[160] Pandya HC, Guller P, Reid F, et al. FRONTIER-4: a phase 2a study to investigate tozorakimab (anti-IL-33 mAb) in COPD. Eur Respir J 2024; 64: Suppl. 68, OA1964. doi:10.1183/13993003.congress-2024.OA1964

[161] Yousuf AJ, Mohammed S, Carr L, et al. Astegolimab, an anti-ST2, in chronic obstructive pulmonary disease (COPD-ST2OP): a phase 2a, placebo-controlled trial. Lancet Respir Med 2022; 10: 469–477. doi:10.1016/S2213-2600(21)00556-7 35339234

[162] Singh D, Brightling CE, Rabe KF, et al. Efficacy and safety of tezepelumab *versus* placebo in adults with moderate to very severe chronic obstructive pulmonary disease (COURSE): a randomised, placebo-controlled, phase 2a trial. Lancet Respir Med 2025; 13: 47–58. doi:10.1016/S2213-2600(24)00324-2 39653044

[163] Kosloski MP, Kalliolias GD, Xu CR, et al. Pharmacokinetics and pharmacodynamics of itepekimab in healthy adults and patients with asthma: phase I first-in-human and first-in-patient trials. Clin Transl Sci 2022; 15: 384–395. doi:10.1111/cts.13157 34523807 PMC8841494

[164] Asrat S, Zhou Y, Rafique A, et al. The high affinity anti-IL-33 antibody itepekimab potently blocks IL-33 induced activation of the ST2/IL-1RAcP signaling complex and inhibits key mediators of airway inflammation. Am J Respir Crit Care Med 2024; 209: A6989.

[165] England E, Rees DG, Scott IC, et al. Tozorakimab (MEDI3506): an anti-IL-33 antibody that inhibits IL-33 signalling *via* ST2 and RAGE/EGFR to reduce inflammation and epithelial dysfunction. Sci Rep 2023; 13: 9825. doi:10.1038/s41598-023-36642-y 37330528 PMC10276851

[166] Vogelmeier CF, Fuhlbrigge A, Jauhiainen A, et al. COPDCompEx: a novel composite endpoint for COPD exacerbations to enable faster clinical development. Respir Med 2020; 173: 106175. doi:10.1016/j.rmed.2020.106175 33032168

[167] Singh D, Guller P, Reid F, et al. A phase 2a trial of the IL-33 mAb tozorakimab in patients with COPD: FRONTIER-4. Eur Respir J 2025; 66: 2402231. doi:10.1183/13993003.02231-2024 40154559 PMC12256803

[168] Ravala SK, Dutka P, Wong M, et al. Structural basis for inhibition of IL-33 induced ST2 activation by astegolimab. Am J Respir Crit Care Med 2025; 211: A6895.

[169] Electronic Medicines Compendium**.** Tezspire (tezepelumab). Summary of Product Characteristics. 2022. Date last accessed: 7 July 2025. www.medicines.org.uk/emc/product/14064/smpc

[170] Rabe KF, Martinez FJ, Bhatt SP, et al. AERIFY-1/2: two phase 3, randomised, controlled trials of itepekimab in former smokers with moderate-to-severe COPD. ERJ Open Res 2024; 10: 00718-2023. doi: 10.1183/23120541.00718-2023 39319046 PMC11417606

[171] Brightling CE, Chalmers JD, Nair P, et al. ALIENTO and ARNASA: study designs of two randomised, double-blind, placebo-controlled trials of astegolimab in patients with COPD. Eur Respir J 2023; 62: Suppl. 67, PA1292. DOI: 10.1183/13993003.congress-2023.PA1292

[172] ClinicalTrials.gov. Efficacy and safety of tozorakimab in symptomatic chronic obstructive pulmonary disease with a history of exacerbations (OBERON). Date last accessed: 2 April 2025. https://clinicaltrials.gov/study/NCT05166889

[173] ClinicalTrials.gov. Efficacy and safety of tozorakimab in symptomatic chronic obstructive pulmonary disease with a history of exacerbations. (TITANIA). Date last accessed: 2 April 2025. https://clinicaltrials.gov/study/NCT05158387

[174] ClinicalTrials.gov. Efficacy and safety of tozorakimab in symptomatic chronic obstructive pulmonary disease with a history of exacerbations (MIRANDA) Date last accessed: 2 April 2025. https://clinicaltrials.gov/study/NCT06040086

[175] Pavord ID, Chanez P, Criner GJ, et al. Mepolizumab for eosinophilic chronic obstructive pulmonary disease. N Engl J Med 2017; 377: 1613–1629. doi:10.1056/NEJMoa1708208 28893134

[176] Pavord ID, Chapman KR, Bafadhel M, et al. Mepolizumab for eosinophil-associated COPD: analysis of METREX and METREO. Int J Chron Obstruct Pulmon Dis 2021; 16: 1755–1770. doi:10.2147/COPD.S294333 34163157 PMC8215850

[177] Rowe SM, Jones I, Dransfield MT, et al. Efficacy and safety of the CFTR potentiator icenticaftor (QBW251) in COPD: results from a phase 2 randomized trial. Int J Chron Obstruct Pulmon Dis 2020; 15: 2399–2409. doi:10.2147/COPD.S257474 33116455 PMC7547289

[178] Kricker JA, Page CP, Gardarsson FR, et al. Nonantimicrobial actions of macrolides: overview and perspectives for future development. Pharmacol Rev 2021; 73: 233–262. doi:10.1124/pharmrev.121.000300 34716226

[179] Shimizu T, Shimizu S, Hattori R, et al. *In vivo* and *in vitro* effects of macrolide antibiotics on mucus secretion in airway epithelial cells. Am J Respir Crit Care Med 2003; 168: 581–587. doi:10.1164/rccm.200212-1437OC 12829454

[180] Mertens TC, Hiemstra PS, Taube C. Azithromycin differentially affects the IL-13-induced expression profile in human bronchial epithelial cells. Pulm Pharmacol Ther 2016; 39: 14–20. doi:10.1016/j.pupt.2016.05.005 27246785

[181] Simoens S, Laekeman G, Decramer M. Preventing COPD exacerbations with macrolides: a review and budget impact analysis. Respir Med 2013; 107: 637–648. doi:10.1016/j.rmed.2012.12.019 23352223

[182] Cazzola M, Rogliani P, Blasi F. Can treatable traits be the approach to addressing the complexity and heterogeneity of COPD? Int J Chron Obstruct Pulmon Dis 2023; 18: 1959–1964. doi:10.2147/COPD.S428391 37705673 PMC10497043

