# Supplementary material for: A translational view of airway epithelial dysfunction in COPD
Source: Eur Respir Rev. 2025 Dec 17;34(178):250110. doi: 10.1183/16000617.0110-2025 (PMC12709363; doi:10.1183/16000617.0110-2025)
Supplement: Supplementary file 1 [file ERR-0110-2025.SUPPLEMENT.pdf]

Supplementary Materials

SUPPLEMENTARY TABLE 1 Potential future therapeutic targets in COPD.

**Cytokines and growth factors**	**Current evidence in COPD**
CCL2	• CCL2 is involved in macrophage recruitment to inflamed tissue [1]<br>• *In vitro* research showed that CCL2-CCR2 may be a potential anti-inflammatory therapy to prevent macrophage accumulation in COPD [2]<br>• *In vivo* research showed predominant CCL2 secretion by bronchial epithelial cells. Increased CCL2 expression was observed in the lungs of mice with COPD, with enhanced macrophage recruitment and activation [3]<br>• In patients with COPD, CCL2 is upregulated to attract inflammatory cells such as macrophages, neutrophils and CD8(+) T-lymphocytes into the airways [1]
IL-6	• IL-6 activates neutrophils, causing neutrophil infiltration at inflammatory sites, which induces the release of elastase and oxygen free radicals, destroying alveolar surfactants, increasing pulmonary vascular permeability and inducing pulmonary oedema [1]<br>• A single-nucleotide polymorphism of IL-6 (174G/C) has been shown to increase the risk of developing COPD [4]<br>• IL-6 increased levels were shown to be associated with worsening in lung function, and mean IL-6 levels correlated with other inflammatory biomarkers (CRP) in patients with stable COPD [5]<br>• IL-6 increased levels have been associated with hospitalisation in patients with COPD [6]
TGF-β1	• Genetic studies have identified single-nucleotide polymorphisms of TGF-β1 associated with COPD [7]<br>• Differential expression of TGF-β1 was observed in the airway epithelium of smokers and those with COPD, as well as decreased expression of inhibitory Smads [8]<br>• TGF-β promoted fibrotic airway remodelling in patients with COPD, further contributing to diminished lung function [9]
TNF-α	• TNF-α has been shown to initiate inflammatory cascades during exacerbations of COPD [1]<br>• TNF-α has been shown to upregulate EGFR expression (implicated in the pathogenesis of COPD [10]) in airway epithelial cells and to stimulate MUC synthesis [1]<br>• A TNF-α antibody (infliximab) and a soluble TNF-α inhibitor (etanercept) have been used to treat inflammatory diseases such as rheumatoid arthritis [11, 12]; the same clinical dose of infliximab used in rheumatoid arthritis has shown no confirmed effect in COPD [11]<br>• The significantly increased incidence of airway tumours and lung infections caused by TNF-α antibodies among COPD patients limits the potential for the investigation of anti-TNF-α treatments in COPD [11]

CCL: c-c motif chemokine ligand; CCR: c-c motif receptor; CD: cluster of differentiation; COPD: chronic obstructive pulmonary disease; CRP: C-reactive protein; EGFR: epidermal growth factor receptor; IL: interleukin; TGF-β1: transforming growth factor beta 1; TNF-α: tumour necrosis factor alpha.

References

1. Wang C, Zhou J, Wang J, *et al.* Progress in the mechanism and targeted drug therapy for COPD. *Signal Transduct Target Ther* 2020; 5: 248.
2. Davies C, Rhodes JA, Barnes P, *et al.* Elevated CCL2 responses in COPD and attenuation by selective chemokine receptor antagonists [abstract]. *European Respiratory Journal* 2015; 46: PA3900.
3. Dong Y, Dong Y, Zhu C, *et al.* Targeting CCL2-CCR2 signaling pathway alleviates macrophage dysfunction in COPD via PI3K-AKT axis. *Cell Commun Signal* 2024; 22: 364.
4. Wu X, Yuan B, Lopez E, *et al.* Gene polymorphisms and chronic obstructive pulmonary disease. *J Cell Mol Med* 2014; 18: 15–26.
5. Lilov A, Marinova D, Slavova Y. The role of serum IL6 in stable COPD patients [abstract]. *European Respiratory Journal* 2018; 52: PA4066.
6. Fermont JM, Masconi KL, Jensen MT, *et al.* Biomarkers and clinical outcomes in COPD: a systematic review and meta-analysis. *Thorax* 2019; 74: 439–446.
7. Yuan C, Chang D, Lu G, *et al.* Genetic polymorphism and chronic obstructive pulmonary disease. *Int J Chron Obstruct Pulmon Dis* 2017; 12: 1385–1393.
8. Aschner Y, Downey GP. Transforming growth factor-beta: master regulator of the respiratory system in health and disease. *Am J Respir Cell Mol Biol* 2016; 54: 647-655.
9. Morty RE, Konigshoff M, Eickelberg O. Transforming growth factor-beta signaling across ages: from distorted lung development to chronic obstructive pulmonary disease. *Proc Am Thorac Soc* 2009; 6: 607-613.
10. Shaykhiev R, Crystal RG. Early events in the pathogenesis of chronic obstructive pulmonary disease. Smoking-induced reprogramming of airway epithelial basal progenitor cells. *Ann Am Thorac Soc* 2014; 11 Suppl 5: S252–258.
11. Rennard SI, Fogarty C, Kelsen S, *et al.* The safety and efficacy of infliximab in moderate to severe chronic obstructive pulmonary disease. *Am J Respir Crit Care Med* 2007; 175: 926–934.
12. Zhang H, Shi N, Diao Z, *et al.* Therapeutic potential of TNFalpha inhibitors in chronic inflammatory disorders: Past and future. *Genes Dis* 2021; 8: 38-47.
